# Towards human-centered AI and robotics to reduce hospital falls: finding opportunities to enhance patient-nurse interactions during toileting

**DOI:** 10.3389/frobt.2024.1295679

**Published:** 2024-01-31

**Authors:** Hannah Rafferty, Cameron Cretaro, Nicholas Arfanis, Andrew Moore, Douglas Pong, Stephanie Tulk Jesso

**Affiliations:** ^1^ Systems Science and Industrial Engineering, SUNY Binghamton, Vestal, NY, United States; ^2^ Human-Centered Mindful Technologies Lab, Systems Science and Industrial Engineering, SUNY Binghamton, Vestal, NY, United States

**Keywords:** human-centered design, robotics, AI, accidental falls, falls prevention, hospital, human factors

## Abstract

**Introduction:** Patients who are hospitalized may be at a higher risk for falling, which can result in additional injuries, longer hospitalizations, and extra cost for healthcare organizations. A frequent context for these falls is when a hospitalized patient needs to use the bathroom. While it is possible that “high-tech” tools like robots and AI applications can help, adopting a human-centered approach and engaging users and other affected stakeholders in the design process can help to maximize benefits and avoid unintended consequences.

**Methods:** Here, we detail our findings from a human-centered design research effort to investigate how the process of toileting a patient can be ameliorated through the application of advanced tools like robots and AI. We engaged healthcare professionals in interviews, focus groups, and a co-creation session in order to recognize common barriers in the toileting process and find opportunities for improvement.

**Results:** In our conversations with participants, who were primarily nurses, we learned that toileting is more than a nuisance for technology to remove through automation. Nurses seem keenly aware and responsive to the physical and emotional pains experienced by patients during the toileting process, and did not see technology as a feasible or welcomed substitute. Instead, nurses wanted tools which supported them in providing this care to their patients. Participants envisioned tools which helped them anticipate and understand patient toileting assistance needs so they could plan to assist at convenient times during their existing workflows. Participants also expressed favorability towards mechanical assistive features which were incorporated into existing equipment to ensure ubiquitous availability when needed without adding additional mass to an already cramped and awkward environment.

**Discussion:** We discovered that the act of toileting served more than one function, and can be viewed as a valuable touchpoint in which nurses can assess, support, and encourage their patients to engage in their own recovery process as they perform a necessary and normal function of life. While we found opportunities for technology to make the process safer and less burdensome for patients and clinical staff alike, we believe that designers should preserve and enhance the therapeutic elements of the nurse-patient interaction rather than eliminate it through automation.

## 1 Introduction

Patients who are admitted to the hospital for major procedures or medical conditions run a higher risk of falling while they are under inpatient care (Hitcho et al., 2004). Injuries resulting from a fall can lengthen hospital stays, complicate recovery, and especially for older individuals, can increase the patients’ risk of dying within the next year (Goldsborough et al., 2019). In addition to these human costs associated with falls, healthcare organizations are motivated to reduce patient falls for financial reasons, as they cost hospitals approximately $50 billion in non-billable expenses annually (Florence et al., 2018). The latest cost of a single inpatient fall, with or without injury, was estimated to be more than $35,000 (Dykes et al., 2023).

Examination of the context most associated with falls and injury offers insights into why this challenge persists. The greatest risk for injury is when a patient falls without assistance, in which no one is present who can catch them or ease their descent to the floor (Staggs et al., 2014). Unassisted patient falls frequently occur surrounding patient bathroom needs, with an estimated 45% of falls related to toileting, and 10%–20% of falls occurring inside the bathroom (Tzeng, 2019). Within the context of in-patient toileting, we noted two major barriers which may present opportunities for technology assistance: 1) the burden of physically lifting and moving patients placed on overloaded, limited nursing staff, who may not be available for bathroom calls promptly on-demand, and 2) social and emotional patient factors which influence the decision to get up and go to the bathroom without asking for assistance (Hitcho et al., 2004; Hignett and Wolf, 2016; Jacobs, 2021; OSHA, 2023).

There are many different types of interventions that already exist to combat this issue, such as 24/7 monitoring (in-person or virtual) and increased frequency in nursing rounding (Goldsborough et al., 2019; Tay and Xie, 2023). Sit-to-stand devices, which assist clinical staff in physically lifting a patient to a standing position are already implemented within hospitals and can improve comfort and reduce occupational injuries (Tang et al., 2017; OSHA, 2023). Solutions such as a gait belt, which is secured around the patient’s torso to provide support to someone who has difficulty standing, and a Hoyer Lift, which is a powered device that lifts patients out of bed to stand them up, have been implemented in healthcare institutions for a long time (Owen et al., 1999); yet these tools are often unused for a variety of reasons (Schoenfisch et al., 2019). Additionally, On the “high-tech” front, Artificial Intelligence (AI) approaches could provide better fall risk predictions (Marschollek et al., 2012; Novin et al., 2021). However, some research has indicated that individuals who are labeled as higher risk of falls may be given less mobility opportunities, which can also lead to poorer outcomes (Capo-Lugo et al., 2023). Assistive robots could reduce the burden on nursing staff by supporting their patient care activities (Pepito and Locsin, 2019; Christoforou et al., 2020). To reduce falls risk surrounding toileting, mobile robots with attached cameras can be used for assessment and telesitting (McFadden, 2018; Oh-Park et al., 2021), and autonomous robotic helpers could be used to lift and move patients to the bathroom with or without nurses present (Wilkinson, 2015; Li et al., 2023). Despite growing enthusiasm for AI and robotics in healthcare, this significant shift must be explored and carefully weighed. Ethics, cost, and whether or not robotic and AI technology is acceptable to clinical staff and patients (Mansouri et al., 2017; Childers and Maggard-Gibbons, 2018; Namba and Yamada, 2018; Saadatzi et al., 2020) can all influence the success and sustainability of a high-tech solutions, and are therefore necessary elements to consider at the beginning and throughout the design and implementation process.

In this paper, we detail a human-centered design research effort to examine clinician and patient needs surrounding the process of toileting, and to explore the potential use of assistive robots and AI aimed at reducing in-patient falls associated with using the bathroom. The intention is to use these early-stage design concepts as the basis for further human-centered design iterations.

## 2 Methods

**FIGURE 1:**
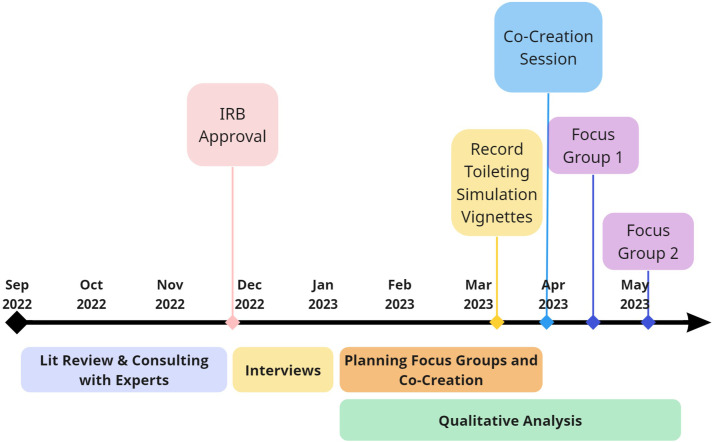
Study Timeline. Research began in September 2022. Over the course of the next year, we planned and carried out a series of human-centered, qualitative research with clinical experts, including Interviews, a Co-Creation Session, and Focus Groups. Qualitative data analysis began after conducting interviews with nurses, which continued throughout the remainder of the research effort and informed how we developed subsequent methods. In spring of 2023, we worked with the Decker College of Nursing and Health Sciences’ Innovation Simulation and Practice Center to develop and film a series of vignettes related to common patient experiences surrounding in-patient toileting, which were shown to participants in the Focus Groups to aid in empathizing with hospitalized patients. The Co-Creation Session and two Focus Groups were conducted between late-March through early May of 2023.

This work was carried out over the course of a year by a team of college seniors as a senior design project, and a faculty advisor in the Industrial and Systems Engineering department from SUNY Binghamton. Students were trained and overseen by the advisor on how to carry out effective and unbiased qualitative research and analysis. The team’s objective was to use human-centered design and human factors principles to explore clinician and patient experiences and needs within the context of toileting in an in-patient hospital setting. AI and robotic technologies were a focal point for the team due to their expanding integration in the healthcare domain. As such, while we did not solely focus on these technologies during our conversations with clinicians, we explicitly prompted discussion around these technologies towards the end of each session, as it is imperative to understand and anticipate the experiences and needs of both clinicians and patients as a result of this shifting paradigm. These insights inform and serve as the basis for early-stage design concepts and use cases in which AI and robotic technologies can assist nursing staff and patients in the toileting process, which is the subject of research which is already underway. Our methods included Interviews, a Co-Creation Session, and Focus Groups, and the overall process is depicted in Figure 1. Participant data was de-identified after collection, and any personal identifiers (e.g., participant names and videos) were stored in secure locations and not shared with anyone outside of the research team. The Binghamton University Institutional Review Board oversaw this work and deemed all research activities as exempt.

### 2.1 Interviews

#### 2.1.1 Participants

To explore the significance of falls, within the context of hospitalization, Semi-Structured Interview s were conducted with five nurses (mean years in practice = 12.8, SD = 11.8). Participants included a registered nurses from med surg oncology, ICU, and ED, a nursing assistant from the ED, and a director of clinical services for home care who was also a nurse by training. Clinicians who assisted patients with toileting as a part of their current (or recent) work routine were considered eligible and recruited through familiarity with the researchers and through snowballing. Although only a small number of these initial interviews were held, they were conducted in order to create a baseline understanding of toileting and to help inform the direction of the future Co-Creation Session and Focus Groups. All participants were informed that they would be compensated for their time and effort with a $50 gift card before they were interviewed.

#### 2.1.2 Materials and procedure

Semi-structured Interviews were conducted online using Zoom software (Zoom, 2023), in which the auto-generated transcript was retained as data. A set of 11 semi-structured interview questions were designed to gather insights related to the overall process of assisting hospitalized patients to the bathroom. This questionnaire assisted in gaining insight into current practices and policies used by organizations to prevent falls, factors causing and contributing to bathroom falls, challenges faced by clinicians in the toileting process, and design opportunities (see Supplemental Material). After verbally consenting to participation, participants were interviewed for approximately an hour. Afterwards, participants were thanked for their participation and compensated with a $50 gift card.

### 2.2 Co-Creation Session

#### 2.2.1 Participants

The second method of data collection was the Co-Creation Session. The participants (*n* = 10, mean years in practice = 3.1, SD = 6.3) consisted of nursing students in the Decker School of Nursing, and a professor in the Decker master’s program with over 20 years of experience. No participants from the Co-Creation Session participated in any other research activity. Participants were recruited through emails, a newsletter, and snowballing. All participants were informed that they would be compensated for their time and effort with a $100 gift card before they took part in the session.

#### 2.2.2 Materials and procedures

Co-Creation Sessions involve a series of activities which build upon each other to generate an abundance of data over the course of one group meeting. Sessions are designed to be fun and open to support the creative process. The session included five activities and asked participants to interact and collaborate to deeply consider challenges and design opportunities surrounding the toileting process. Participants also designed their own “ideal solutions” which could aid them in the process of patient toileting, which served as examples of technology which directly addressed pertinent challenges in ways that were deemed appropriate for the highly specialized context. The session guide can be found in the appendix.

The session was conducted in person in a large meeting room on the SUNY Binghamton campus. Two team members facilitated the session, while the others took notes and provided additional supplies when requested. Audio and video recordings were collected to capture participants’ responses and conversations which might be missed while observing the session. Participants first read and signed an informed consent form to participate and allow us to record the session. Facilitators then described an overview of what to expect during the session (see Supplemental Material detailed session outline).

**FIGURE 2:**
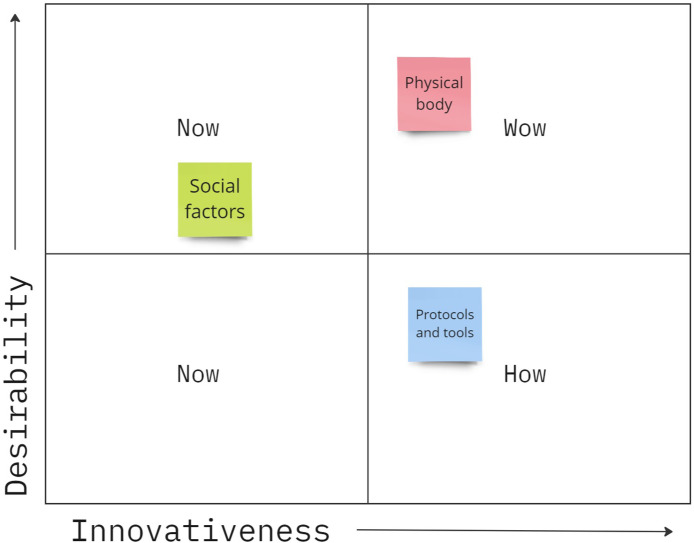
Example How-Now-Wow map. Ideas categorized as “Now” concepts were designs that exist today, “How” concepts were innovative, but had low desirability so it was hard to see “How” they would help, and “Wow” concepts were both innovative and desirable. Post-it notes were color coded to show social factors (green), things related to the physical body (pink), or processes and tools used in toileting (blue).

Part one involved two activities to rapidly ideate and then organize a broad sample of concepts related to the current process of in-patient toileting, challenges faced, and potential solutions to challenges. First, participants responded to a series of prompts to generate a wide range of ideas pertinent to the context of toileting. Each idea was written on a post-it note. Post-it notes were color coded according to three themes: social factors (green), the physical body (pink) of both patients and nursing staff, and various protocols or tools used in the toileting process (blue). Participants were encouraged to have conversations with other participants to generate a fuller set of ideas. Participants next worked in small teams to talk about each concept and categorize them into a How-Now-Wow map (Figure 2). In considering how individual concepts could be used to assist in the process of in-patient toileting, participants were instructed to place post-it concepts anywhere within quadrants of a graph representing categories of (1) “Now,” or tools/processes that currently exist and were therefore not highly innovative, (2) “How,” or concepts that were innovative, but were not highly desirable or practical, therefore were hard to understand “how” they may help, and (3) “Wow,” or concepts that were seen as both innovative and desirable. Afterwards, each team delegated a leader who summarized their discussion and presented the map they created to the rest of the group.

These initial activities served to prime a creative task in which participants designed solutions to what they felt were the most pressing challenges they and their patients experienced during toileting. In the second part of the session, participants were instructed to individually design what they would consider to be an ideal tool and process to assist them in the context of in-patient toileting. They were provided creative supplies and encouraged to design and sketch and present their designs, while other participants anonymously rated each design. After 30 min had passed, participants then “pitched” their design to the rest of the group, briefly describing what they designed, how it was used, and what challenges it was designed to address. All participants then quantitatively rated each design (including their own) using a Likert survey to indicate the extent to which each design was helpful, respectful of patient privacy, how easy it would be to implement the tool within a hospital, how desirable the solution was, and perceived ease of use.

Finally, the entire group convened and were asked a semi-structured series of questions. First, participants were instructed to think about a world 100 years into the future, in which robots and AI might be commonplace, and discuss how they thought this technology would be used in healthcare, the impacts on patients, and what an ideal future of in-patient toileting might look like. Lastly, participants were asked if they had any concluding thoughts they wanted to discuss. The session lasted 2 hours in total, and afterwards participants were thanked and compensated with a $100 gift card.

### 2.3 Focus Groups

#### 2.3.1 Participants

The third data collection method was to use Focus Groups to explore toileting needs and challenges from a patient’s perspective. Clinicians were again recruited to participate due to their intimate knowledge of the clinical environment. Two sessions were held, with a total of seven participants (mean years in practice = 18.8, SD = 4.97), which included four RNs, a physical therapist, a phlebotomist, and a pharmacist, some of whom were currently in leadership and education roles. No participants from the Focus Groups participated in any other research activity. Participants were recruited through familiarity with the researchers and through snowballing. All participants were informed that they would be compensated for their time and effort with a $50 gift card before they took part in the Focus Group.

#### 2.3.2 Materials and procedure

Participants were shown a series of filmed vignettes to prime them to think about challenges and desires from a patient’s perspective. Each scenario was designed around some sort of awkward, uncomfortable, or socially compromising situation, and participants were instructed to place themselves in the shoes of the patients in the videos.

The vignettes were created through insights from the Phase 1 interviews and collaboration with domain experts. Three scenarios were designed in which hospitalized patients experienced challenges when needing to use the bathroom. The Director of Simulation and Practice at the Decker College of Nursing and the Decker Video Production Leader assisted researchers in refining realistic scenarios, and in filming and editing these down to brief vignettes. A description of the three vignettes is presented in Table 1 (see Supplemental Material for a full description and Focus Group questions). During the Focus Group, participants were asked semi-structured questions related to the patient experience of toileting in a hospital, and were shown all three vignettes in the same order. After each video was shown, the participants reflected on the videos and gave input and feedback on what could be changed, and how the situation could be improved for the patient. The session lasted 1 hour and participants were compensated with a $50 gift card.

**TABLE 1:** Scenarios developed for Focus Groups. See Supplemental Material for a full description and Focus Group questions.

Vignette	Scenario description
1st	The patient feels dizzy and unaware of their own limitations. The patient tries to get up to use the bathroom, but fell on the floor and was later discovered by a nurse
2nd	A young, injured patient needs to use the bathroom, but does not want to inconvenience the nurses. When the patient finally calls for help, the nurse does not immediately respond to the call bell, which causes the patient to feel frustrated by their lack of autonomy due to the injury and experience a downward emotional spiral. When the nurse finally arrives, the patient acts irritated
3rd	An older patient is forced to use a bedpan instead of the bathroom. The older woman (played by a simulation dummy) is being assisted by two younger male nurses who are not sensitive to the patient’s urgency, which fuels embarrassment. As the nurses are helping the patient use the bedpan, cleaning staff and the patient’s family walk in, causing additional embarrassment

### 2.4 Analysis

Qualitative data collected through the interviews, Co-Creation Session, and Focus Groups were analyzed through content analysis (Downe-Wamboldt, 1992). This data consisted of transcriptions from Interviews, Focus Groups, and the Co-Creation Session (transcribed manually from videos), written notes, design drawings and explanations. Google Sheets was used to code data, tabulate counts and calculate inter-rater reliability. The team worked iteratively to review data and create bottom-up derived recurring concepts (i.e., codes) to better understand and interpret data. An initial code book was then created after review and deep discussion among the team, consisting of individual codes and a descriptive definition of what the code meant. To validate that all team members interpreted the definitions similarly and applied the same codes to the same situations, a subset of data was used to check agreement and interrater reliability using Cohen’s Kappa (McHugh, 2012) and percent agreement. The team continued to work iteratively to refine codes and discuss deeper meaning and overarching themes.

The valence of statements made by participants (i.e., the pleasantness or unpleasantness of a concept expressed by participants; (Frijda, 1986) were also recorded by raters. Every statement was subjectively assessed as being positive (+1), negative (−1), or neutral (0) by raters, allowing for an examination of the positive and negative associations for coded concepts. This aided in identifying common challenges and design opportunities from the collected data.

The count of each code was compiled into a bar graph to display how often each code was utilized. The average valence of each code was also put into a bar graph to compare the valences of the codes. Quantitative data from Co-Creation Session surveys was described through descriptive statistics and related back to the designs generated by participants in the session. Information collected in each of these stages was reviewed by the team to inform the proceeding methods of data collection.

## 3 Results

### 3.1 Overview of findings

**FIGURE 3:**
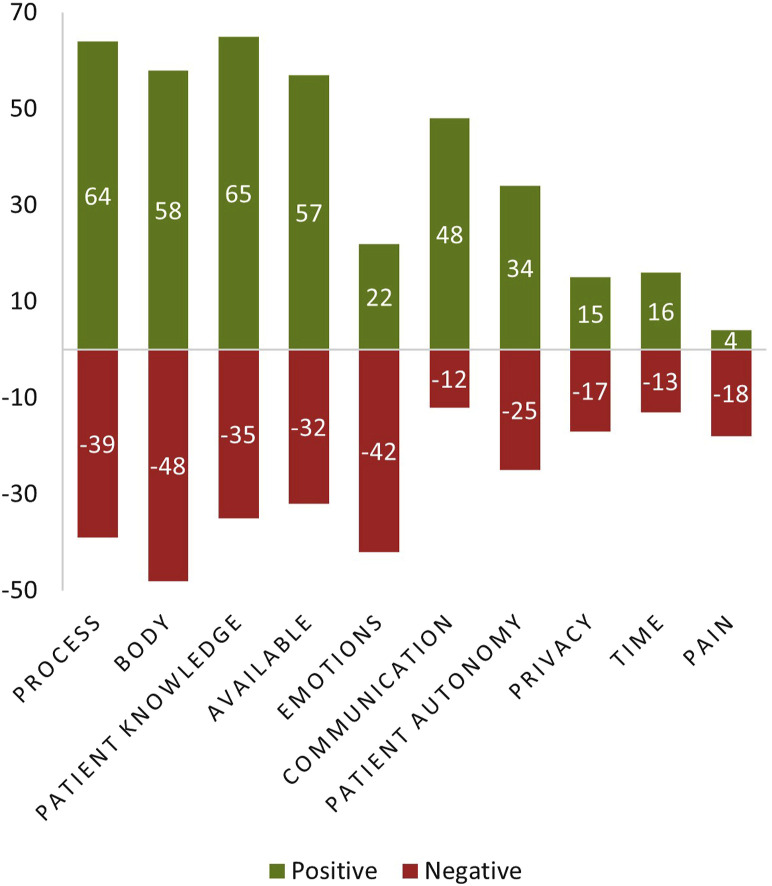
Counts of Positive and Negative Statements Associated with Each Code. Individual codes, which were iteratively developed by researchers through qualitative analysis, are presented on the X Axis. Each coded statement was also qualitatively assigned a positive or negative valence if raters believed that the statement made by participants was a positive or negative expression. The total count of coded statements which had a non-neutral coded valence (positive or negative), is labeled and graphed on the Y Axis. Codes are also sorted according to overall counts (positive and negative), such that the most frequently occurring code (Process) is presented on the left, and the least frequently occurring code (Pain) is presented on the right.

The team iteratively developed an initial set of six codes and applied these to a small subset of the data in order to establish agreement and check inter-rater reliability using Cohen’s Kappa (see Supplemental Material). Through continued iterative application and refinement, 10 final codes were established and applied to all data, resulting in a total of 857 applied codes. Codes related to the process of toileting (*n* = 136), the physical body (*n* = 134), knowledge of patients and their risk factors (*n* = 130), availability of tools, resources, and space needed (*n* = 120), emotions/feelings of patients (*n* = 83), communication (*n* = 81), patient autonomy (*n* = 71), privacy (*n* = 42), timelines/demands (*n* = 37), and pain to patients or nursing staff (*n* = 23). Figure 3 shows the total count of statements associated with each code with respect to coded valence (positive and negative) for non-neutral valence statements. The final codes, definitions, representative quotes, counts, and percent agreement, along with much of the visual data from the Co-Creation Session, can be found in the Supplemental Material. In the following sections, we discuss our findings in greater detail and what we learned about the process of toileting, current barriers, and opportunities for technology to assist patients and nursing staff, particularly nurses and nursing aids.

### 3.2 What does a normal toileting process look like, and why is it challenging?

While hospitalized, some patients need assistance from staff to get up and to move. Even if a patient is physically capable of getting up and walking, they still may be required to ask for assistance. In an in-patient setting, one participant indicated that patients typically have: “*ambulation orders in place from the doctor*”, in which patients are *“either in bed–you have ‘bed rest’ ordered, so they do not want you to get up, for whatever reason, by the physician. Or you can ‘ambulate with assistance’, or you can ‘ambulate independently’” (Nurse, Interview 5),* and noted that such orders are not typically placed for patients in the emergency department, even if their stay within the ED is prolonged due to bed availability. Patients required to “ambulate with assistance” are instructed to press their call bell to alert clinical staff to their needs, most frequently nursing staff like registered nurses (RNs), licensed practical nurses (LPNs), or nursing aids (NAs), and must wait for assistance to get up and use the bathroom in order to reduce the risk of falling.

**FIGURE 4:**
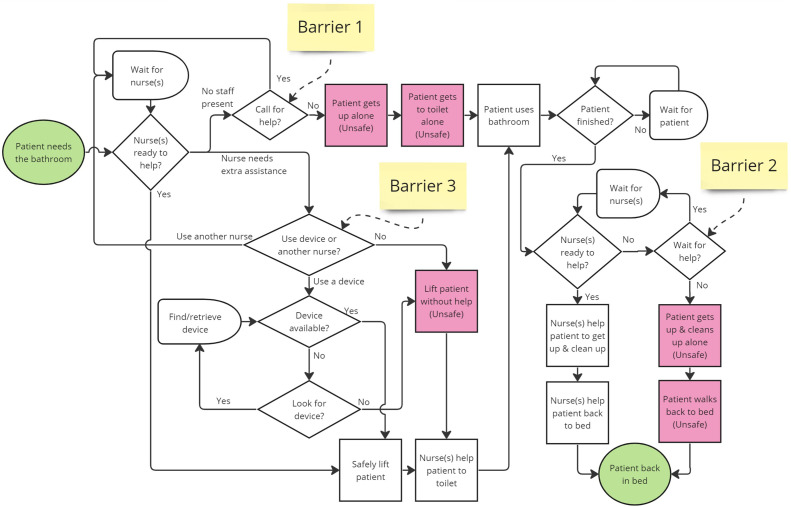
Process map for taking patients to the bathroom. Three Barriers are labeled, which contribute to patient and staff decisions to engage in unsafe behavior. The process starts when the patient needs to use the bathroom. If a nurse is not present at the onset or when they are finished in the bathroom, they can call for assistance using a call bell, or they can choose to get up alone (Barriers 1 & 2). If a nurse responds to the call, or the patient needs the bathroom during rounding, the nurse will determine if they can help the patient, or if they need extra assistance by asking another nurse (or more), and/or retrieve and use a mobility assistive device. The nurse feels that they cannot afford these delays, they may choose to lift the patient alone, and risk an occupational injury or having the patient fall (Barrier 3).

For patients who need assistance in toileting, we asked participants to describe the process of getting a patient to the bathroom, and constructed a process map based on their statements (shown in Figure 4). We will first discuss this process in general, and delve further into the barriers and technology considerations for staff and patients in the following sections.

In the context of toileting, patients can fall at any stage of the process, but frequently, *“when people fall, they fall from the bed to the floor. They fall from the second they stand up” (Nurse, Interview 1)*. Another participant stated: *“We see a lot of people that fall getting off of the toilet because they hold on to something, and they’re standing, and they follow over, or as they’re standing up, they get dizzy or light headed” (Nurse, Interview 5)*. For this reason, it is important that staff be present from the very beginning of the toileting process and when the patient gets up in the bathroom. When a patient needs to use the bathroom, they can wait for nurses to visit and ask to use the bathroom then, or they can alert staff to their needs by pressing a call bell and waiting for their nurse to take them to or from the bathroom. Importantly, patients do not always ask or wait for help for many reasons including urgency of toileting needs and not wanting staff to assist during the toileting process (see Barriers 1 & 2 on Figure 4, discussed more thoroughly in following sections).

For nursing staff, the process of getting the patient to the bathroom is physically involved and takes nursing time: *“It can be a 20–30 min process from bed to bathroom to waiting then back to bed, and there are other tasks that need to get done” (Nurse, Interview 2)*. The average time for toileting a patient was estimated at 30 min by other participants as well. Some patients require assistance from more than one staff member: *“[When getting them out of bed], if they’re able to sit themselves up, then they will. If not, it could be a two-man job” (Nurse, Interview 1)*, which presents additional challenges when having to find and wait for the second person. Time limitations and staffing shortages are discussed more thoroughly in the next section, as this is a key barrier in the toileting process. If nurse(s) need additional aid for heavier and/or weaker patients, they may also use mobility assistive equipment to lift and move patients to make the process safer and more ergonomic for nursing staff, and/or maintain contact while the patient moves to help guide or catch the patient if they fall. Participants noted many limitations of these tools, including the difficulty of use or unavailability when needed, causing a delay. Delays in finding other staff members and/or mobility assistive devices can lead a nurse to decide to unsafely lift the patient alone (see Barrier 3 in Figure 4). After lifting a patient up and getting them on their feet, nurses also assist unsteady patient in walking to and from the bathroom. For nursing staff, applying their expert judgment to decide how to assist the patient can be a complex cognitive task. When a patient’s mobility status is ambiguous, assisting them can require constant reassessment and readjustment over a prolonged interaction. One participant described this involved process at length:

So they have to meet certain criteria to be able to walk. You have to be conscious and alert and oriented enough, and able to follow commands… then I will actually try to get them to the side of the bed, dangle them with good feet, and see how they feel…I have them stand, and I usually have them get their bearings and get their composure, make sure they’re able to handle their own body weight with my assistance or with the device [e.g., walker or cane], whatever they need… And I’ll continuously assess them. Kinda ask them how they’re doing. Are you having any symptoms? Is anything bothering you as we’re walking and as we’re taking a few steps. Then I have a better idea of, hey, do I think this person has the capability of walking all the way to the bathroom at the end of the hallway… or are they kind of weak, or are they kind of unsteady? And maybe we should just turn them around. Bring them back to the stretcher in their room (Nurse, Interview 5).

The assistance of an unsteady patient involves continuous assessment, communication, cooperation between patients and nurses, and an ability to quickly readjust to avoid a fall. The presence and assistance of nursing staff makes the process of toileting safer for patients, but the overall process can be improved to enhance efficiency and safety. Technology may be used to ameliorate this process, but contextual and human factors should be carefully considered during the design process.

### 3.3 Staff barriers and technology considerations

Since patients who need the bathroom are at risk the moment they decide to get out of bed, nursing staff need to identify which patients are at a higher risk to prioritize resources and be prepared if the patient decides not to call for assistance. Most participants mentioned that fall risk is estimated and recorded upon admission through a falls risk assessment, involving: “*a series of questions that you answer upon [patient] triage upon their intake to the hospital, and depending upon how many checkboxes you get you’ll get a low, moderate, or high fall risk assigned to you.” (Nurse, Interview 5)*. However, one noted that: *“A MAHC-10 [fall risk] assessment is done, but mostly everyone scored high enough and was deemed fall risk.” (Nurse, Interview 2)*, in which case, the score would provide limited insight. If patients are at high enough risk, a person can “sit” in the patient’s room to watch them, but this is highly dependent on staff availability. Hospitals can also use pressure sensor alarms or “telesitters” to monitor patients through a camera and alert staff when someone is getting up, but this may not provide enough advanced warning. One participant noted that with bed alarms and chair alarms, “*nurses go running when it rings*”, because it alarms when the patient is already getting up and the nurse must rush to intervene (Nurse, Interview 3). Participants also indicated that actual fall risk constantly fluctuates due to changes in health conditions and/or status in the moment someone gets up. Participants noted various transient conditions that increase a patient’s fall risk, such as bacterial or viral infections (Focus group 1), various medications taken in the hospital such as narcotics (Interview 2, Focus group 1), “sundowning” (Khachiyants et al., 2011), in which “*people with Alzheimer’s or dementia, or memory impairments basically, become more confused at sundown time.*” (Focus group 1); “orthostatic hypotension”, where *“sitting up or standing too fast causes blood pressure to drop and causes dizziness and lightheadedness in a patient*” (Nurse, Interview 2). Also changes in blood sugar if the patient is not eating well (Focus group 1), or muscle becoming “deconditioned” while a patient sits in bed for a day or longer (Focus group 2). All Interview ees perceived that elderly patients are at greater risk of falls and injury due in part to increased frequency of these conditions. While nursing staff may have access to the assigned fall risk scores, the patients’ true fall risk must be constantly reassessed due to these fluctuations, and requires, *“nursing judgment at the time of movement” (Nurse, Interview 2)* to make decisions about how the patient will be assisted, and how to reassess and adjust during the lifting and moving process. However, nurses with less experience may be uncomfortable in relying on their own judgment. During the Co-Creation session, two younger nurses discussed how, “*every time I get to the patient’s room, and they’re like, ‘can you help me to the bathroom?’, and I’m like, ‘I don’t know’” (Nurse, Co-Creation),* and discussed their desire for more prominent displays of risk and assistance needed to help them navigate the ambiguity. These discussions showed us that risk prediction quality and reflection of changes in real-time can be improved upon to provide insight to nurses.

As discussed previously, for nursing staff, the process of bringing a patient to the bathroom takes a lot of time and physical effort. During Interview s and Focus Groups, when directly asked about the most challenging aspect of the toileting process for nurses, participants named low staffing and time constraints (Interview 2, Focus group 2) as well as interruptions during other work (Focus Groups 1), physical strain on the body (Interviews 1 and 4), awkwardness of equipment (Interview 3) and room size/arrangement (Focus Group 1) as their top complaints.

For busy nursing staff, the interruption of workflow due to bathroom calls can be frustrating. One participant stated that, “*Yes, [technology could be helpful] because [toileting] takes a lot of nurses time. Nurses dread getting bathroom calls” (Nurse, Interview 1)*. When asked how new assistive technology could affect her job responsibilities, she added:

[toileting] would be done quicker and save a lot of time. People wouldn’t dread doing it as much. If it is a 2 person job [to assist the patient] - that’s an issue. An assistive device to help with one nurse instead of having her go find another one. Nurses get mad and patients get mad for waiting. (Nurse, Interview 1)

In the second Focus Group, another participant described how time was a challenge because: *“There’s competing tasks that you have to do. And then typically taking somebody to the bathroom is very time consuming* … *I don't want to say it's a hassle, but it's essentially just–it's a bigger task than it seems” (Focus group 2)*. Another participant then added that while a patient is in the bathroom: *“we as nurses will say, ‘Okay, in that minute I’m going to go and do something else’, because we have so many tasks, and then they’ll fall in the meantime, because they’re not waiting for us to come back” (Focus Group 2).* While a nurse can recoup some time and perform other work if they do not need to be present, participants recognized that leaving the patient alone introduced additional risks, and this risk might remain regardless of the presence of assistive technology. One participant noted how, when considering if technology could be used for toileting: *“It’s a hard question, because accidents do happen in the bathroom if a nurse is not attending, but [technology] would be valuable due to staff shortages.” (Nurse, Interview 2)*. She later added, *“Hospitals are doing whatever they have to do to fill staffing needs, and technology of any kind would help reduce the stress and workload on nurses*.” Staffing issues may get worse as a result of the “*Silver Tsunami*” in which a large number of “*baby boomers are aging out*” and will soon need more medical assistance (Nurse, Co-Creation). When nursing staff are overwhelmed, it can lead to burnout, and even transfer aggression towards the patient:

I think, also, another thing that we don't talk about with this–which is a real ethical problem– is that, and I'm trying to say this as not judgmentally as possible–but literally, there's fatigue. So what happens is the nurse is like, ‘No, I'm not going there. I've been there 10 times. I'm done. I'm not going back to that room’, and that's wrong. That's wrong, you know, to punish a patient. Nobody sets out to do that, but that's what happens… so we have these real ethical conundrums where, you know, we're not responding how we should, because we're tired. We're overwhelmed. We have 70,000 other things to do, and we're also, you know, misplacing our own frustration on the patient who is calling for the tenth time or twentieth time, or whatever. (Nurse, Focus Group 2).

Nurse exhaustion and burnout can have significant consequences for patient safety and moral treatment. The risks and opportunities introduced through new technology need to be considered within the current reality of healthcare, in which burnout and exhaustion put clinical empathy at risk (Anzaldua and Halpern, 2021).

Multiple participants noted the difficulty in lifting and moving patients and the risk of occupational injury: *“[something I struggle with most is] if they’re really heavy, it’s very hard to fully lift them. This may strain the nurse. Also, getting them from bed position and lifting them off the toilet [are physically challenging]” (Nurse, Interview 1)*. Unfortunately, time pressures can affect the decision to engage in an unsafe lift, and one experienced nurse discussed how this led to her own occupational injuries:

I think also keeping in mind the nurse’s physical abilities…I remember I hurt my back a few times as a nurse getting people to the bathroom, and especially if you can't– because it's such a timely thing that if you can't find [a second nurse to assist] right away, you're trying to get them by yourself. (Nurse, Focus Group 2).

Physical assistive devices can be used to lift patients to make the process safer and more ergonomic for nursing staff, and/or maintain contact while the patient moves to help guide or catch the patient if they fall. While participants were familiar with devices already in use to avoid these injuries, such as the Sara Steady, Sit-to-Stand, Hoyer lift, and “gait belts”, they had many criticisms; sit to stand devices are *“bulky”*, and still require *“at least two people to assist and move the patient” (NA, Interview 4)*. A common complaint was that the devices were large and cumbersome to operate in small spaces: “*Hospital rooms can never seem to be big enough… Rooms aren't getting bigger and the amount of equipment that we’re using is increasing… Depending on what’s going on for each patient, that can make navigating that equipment problematic.” (Focus group 1)*. With all of the equipment in the room, the nursing staff are put in awkward postures: “*we have to move our bodies in weird ways just to make sure they’re safe when they’re getting [to the bathroom]”,* and this increases the risk of occupational injury (Nurse, Co-Creation). Another participant noted that in cramped hospital bathrooms: *“if you can't get a machine, or a sensor, or a cane, or a walker to physically be accommodated in the space that they’re in, that’s a vulnerability point” (Nurse, Interview 5)*. Another challenge was that these tools are frequently unavailable when needed. One participant noted:

We have some pretty amazing tools for mobility, but then the hospital only buys like one. No one's going to the stockroom to get them, they're just using what they've got at hand… if you can't see them in the room, you're just going to grab the patient up. (Nurse, Co-Creation)

If devices are not available when they are needed, they cannot be utilized to prevent staff injury or reduce falls. Another participant imagined that if designers *“made [lifting devices] fold up and compact in every room… like walkers are in every room. Every bed has a walker”*, this could ensure that devices were available to be used when needed (Nurse, Co-Creation).

Importantly, multiple participants mentioned that using lifting equipment can cause a patient pain: *“I know that [the Hoyer lift] can be really painful. There was one time where I had a patient, like, screaming, crying while using the Hoyer” (Nurse, Co-Creation)*. Another participant then considered if patients could be given pain medications prior to using a lift device, but noted *“you’d have to give the medication 30 min before using the bathroom”*, which would make it challenging given the unpredictability of bathroom needs.

These critiques offer important insights when considering designs of assistive tools. Nursing staff might benefit from technology that frees up their time to focus on other tasks and reduces their physical strain in lifting and moving patients, but our participants identified constraints which require attention in design. Robotic and AI applications will present new complications and risks, and the opportunity for social support during painful moments of care may be eliminated.

### 3.4 Why do patients risk falling to use the bathroom, and what role can technology play?

One reason patients get up without assistance is directly related to nursing staff and timing challenges, which can cause delays in reaching patients in a timely manner. One nurse participant recalled a recent experience she had while hospitalized after a surgery:

I had to go. I had multiple bags of fluid, had [pressed the call bell for assistance]. I was like, you know, I have to go, and I waited and waited, and finally I got up with my drip [still connected to IV pole], and you know, went to the bathroom myself. Luckily I was, you know, I did dangle [my legs] for a few minutes before getting out of bed, but I was back in bed by the time they came. So the time factor was huge, and I was annoyed and resentful that they didn't come. (Nurse, Focus Group 2).

While this experienced nurse was well aware of the risks, the urgency of need led her to decide not to wait, and she felt upset with staff. Patients can also perceive a social pressure to not request assistance to avoid interrupting staff. Patients might “*feel guilty”* for asking for help because they worry about, “*taking away resources in an already short-staffed area” (Participant 2, Focus Group).* Another participant noted that patients are: *“acutely aware of what’s going on around them in terms of staff”,* and, *“They’re tuned-in in a way that they shouldn't be. They shouldn't be responsible for what’s going on, or feel accountable” (Nurse, Focus Group 2)*. This social dynamic may constitute another route by which patients can suffer when hospitals are understaffed. Nursing staffing and time pressures are challenging and frustrating for staff, but patients who also experience this stress may engage in unsafe behaviors under this perceived social pressure to avoid being a nuisance.

Given the myriad limitations experienced by nursing staff and ambiguity over whether patients can be safely assisted to the bathroom when they need to go, even patients who can get up are sometimes required to use other means. Aside from the bedside commodes, in which a bedpan is attached under a portable seat which can be placed close to a patient bed or chair, patients can also use a bedside urinal (for women or men), bedpans, catheter or other similar devices such as a “PureWick”, or “chucks”, which are absorbent pads that are placed underneath a patient. These options can provide convenience to nursing staff: “*having a patient stay in bed as much as you can, is probably easier for nurses… [because] it's way harder and more effort [to get patients up]”*, but the convenience can come at a cost to the patient, and this participant noted, *“when you think about it, patient care is the number one priority”. (Focus Group 1)*. The psychological discomfort experienced by patients as a result of these alternative means was expressed by multiple participants. Especially for patients who feel capable of getting up, being forced to use these methods can be humiliating and harmful:

[Having patients use chucks or a bedpan is] another way that we dehumanize and that's such a source of stress and psychological agita. You know, like we've just dehumanized you [during medical processes], now go poop and pee in your bed. What does that, what is that about? … I have patients that will *so* not want to engage with all this that they've become constipated. And then we now have another huge issue, like they will hurt themselves to maintain their dignity or comfort or independence, or whatever it is that they're trying to protect. (Nurse, Focus Group 2).

Another participant then added that since many hospital rooms are shared, voiding in bed in the presence of other patients is *“another source for embarrassment on multiple levels. So it's like they’re better off trying to go by themselves or to sneak off [to use the bathroom]” (Nurse, Focus Group 2)*. Trying to avoid this embarrassment–which may be a direct consequence of calling for assistance–can motivate patients to risk getting up and toileting on their own. Participants also discussed the detrimental physiological impacts of staying in bed for too long, such as pressure injuries, which can be caused or exacerbated by a bedpan, and loss of independence when a patient becomes weaker from muscle atrophy. They noted that some patients become unmotivated to get out of bed over the course of hospitalization. One participant expressed that with technology to enable easier voiding in bed: “*if the patient had no motivation to get out of bed, it’s even less motivation” (Nurse, Co-Creation)*. Nurses play an important role in encouraging patients to get up and engage in their own recovery, and going to the bathroom can be therapeutic. After viewing a vignette of a female patient using a bedpan during a Focus Group, participants described the importance of giving patients the option to get up and use the bathroom:

Participant 1: It gives [a patient] some type of confidence that she's not just, I hate to use this phrase, but like rotting in [her own waste]. Like she's not just sitting there in a bed waiting for someone to help her with every aspect of her life. She feels like she's still human and it's her own body.

Participant 2: Yeah, that’s a good point … If someone is capable of doing things, you know, in a safe way with or without assistance, you want to encourage that independence too because that's important for maintaining dignity and all the other kind of psychological things that go with that. (Focus Group 1).

Overall, our participants indicated that nurses recognize the patients’ desire for autonomy and privacy when toileting, but this can be a point of contention when the patient requires nursing assistance. Participants noted that the choice to get up without assistance can be: “a *pride thing. And they don’t want to give up their independence, they also don’t want to admit that they can’t do it.” (Focus group 1)*. Another participant said: *“the pride thing is a very common thing that I’ll see with elderly folks…You have to say, ‘Hey, it's okay. I’m here to help you’, because they physically just can't do it” (Nurse, Interview 5)*. When a patient doesn’t want assistance, the *social communication* that it’s alright to ask for help is necessary to establish cooperation and recognition of the patient’s limitations. The same participant later stated: “*if you have technology to take a patient to the bathroom, a lot of times patients don’t use it. So they may have access to a walker, a cane, and they don’t use it because they think they don’t need it*”. While some other participants noted that robots could be a desirable replacement to a nurse in the context of toileting, “*because a robot is not going to judge you” (Nurse, Focus Group 2)*, the ability to convince the patient to accept help is a human quality, and current technologies do not provide this type of function.

Feeling frustrated by not getting assistance the moment it is needed, or feeling guilty for asking for assistance, coupled with the embarrassment of needing assistance in going to the bathroom creates a social and emotional pressure on patients to not ask for help. We believe this goes a long way in explaining why patients take risks and fall in the hospital. However, when patients need assistance, nurses provide physical *and* emotional support when getting the patient out of bed and walking, and therefore the entire toileting process could be viewed as a form of therapy. A nurse’s ability to be attuned to a patient’s physical and emotional condition enables them to assess the patient and encourage them to engage in their own recovery. This beneficial patient-nurse touchpoint should be recognized when considering any technology that may alter or eliminate the human-human interaction.

### 3.5 Assistive technologies envisioned by nurses and perceived tradeoffs

The Co-Creation Session was our most prominent source of information in considering what innovative technologies might do to improve the process. A summary of participants’ designs is seen in Table 2. Drawings of these designs along with other visual data from the Co-Creation Session is available in our Supplemental Material.

**TABLE 2:** Participant designs to assist in the toileting process created in the Co-Creation Session. Descriptions were provided by participants and summarized by researchers. Researchers also qualitatively categorized each design according to what type of device that was described.

Design	Description	Type of device
Potty Alert	A call bell with multiple added functions. The nurses’ station would hear different chimes for bathroom requests, so they would understand what the patient needs	Communication/information
Mobility Bands	Color coded band to allow nurses to easily identify how many nurses are need to assist the patient when they are moved	Communication/information
Smart Bell	This design has a phone-like function so that the patient would be able to communicate directly with the nurses station. There are also additional buttons for hunger, thirst, etc. to help the nurse understand what the patient needs and help reduce the number of trips the nurses have to take to help the patient	Communication/information
Urotech	This design would attach wireless sensors onto a patient when they are admitted to the hospital. This would help to give a baseline reading of the patient to see how often their bladder fills up and they have to go to the bathroom. The readings would go directly to the nurse’s phones and monitors to track the patients. It would also notify the nurses when the patient’s bladder is close to full, so they can help the patient before there is an accident. This design would also analyze fall risk scores periodically and determine individual patient trends	Communication/information
Toilet Tool	A mobile application and a tool that interfaces in the nurse’s computer. It would display the patient’s date of birth, mobility status, how they ambulate, if they have been evaluated by physical therapists, how independent they are, and what languages they know. It would primarily be used for accessing patient data quickly and efficiently to know how to assist them during toileting	Communication/information
Partial Automated Weight Bearing Lift	Combination between a walker and Hoyer lift device. It would also detach and help take the patient all of the way to the bathroom. Alleviates pressure and stress from the nurse, since moving the patient to the correct position can potentially hurt the nurse	Lift/mobility assistance
Walker Backing	Gives the patients a support to either sit or lean on and is based on a standard walker. Sturdy flaps would rotate in from the sides of the device and come underneath the patient, which would provide support in case they fall backwards	Lift/mobility assistance
Mobile Bed	Similar to a normal patient bed but has a head that turns 90°. Nurses can remove the lower half of the bed, converting it into a commode. The entire process would be overseen by a nurse. It has a treadmill feature that would slide the patient down to the edge of the bed for easier movement	Lift/mobility assistance
Seated Bed	The patient’s bed would have three moving parts to better assist nurses in getting a patient to a sitting position	Lift/mobility assistance
The Shifter	Mechanical device helps move a patient who is in the bed onto their side, which would expose their backside to eliminate the patient’s need to turn	Lift/mobility assistance
Adaptive Seat	The design involves attachments to either a toilet or commode. The seat would bring the patient to an optimal height that would be most comfortable and avoid needing to “drop” the patient onto the toilet. It would also be able to tilt in multiple degrees of freedom	Lift/mobility assistance

All 11 participant designs could be broadly categorized into two categories. Participants designed tools which assisted with understanding patient toileting needs through better communication and/or collecting and displaying information (5/11). This information could be used to improve how a patient was assisted and allow nurses to bring patients to the bathroom at more opportune moments, before the need to toilet became urgent or disruptive. These applications are most relevant when considering how AI tools can be created to assist in the toileting process. Participants also designed tools that assisted in lifting and/or moving patients (6/11), reiterating the necessity to reduce the physical effort and the risk of occupational injury when assisting the patient. Five of these six were redesigns or extensions of existing ubiquitous equipment rather than the addition of a new tool, including hospital beds (“Mobile Bed”, “Seated Bed”), walkers (“Partially Automated Weight-Bearing Lift”, “Walker Backing”), and toilet seats (“Adaptive Seat”). As discussed above, nursing staff can struggle with bulky mobility assistive equipment and may not seek out tools which were not available at hand when needed, and the design of tools which would be present in every room without added to the clutter provided some additional confirmation. Insights from these designs are most relevant when considering how robotic applications could be used in toileting.

**FIGURE 5:**
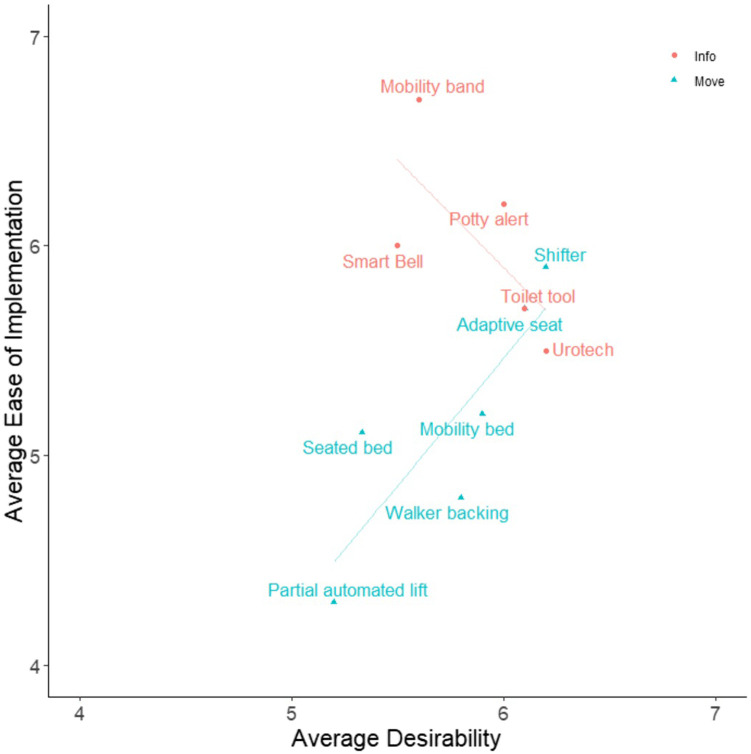
Average Desirability vs. Average Ease of Implementation for Participant Designs. Participants reported their own perception of Desirability and Ease of Implementation (along with 3 other qualities) on 7 point Likert scales for each participant design (including their own) created during the Co-Creation Session. Averages of these measures for each design are presented in the figure. Designs were qualitatively categorized into one of two groups: tools which provide additional patient information (Info), or tools which aid in moving patients (Move) to assist clinicians in the toileting process. Regression lines of best fit were added to the figures for illustrative purposes, though statistical analyses were not conducted due to the limited sample size. Further information on each design is presented in Table 2 and the Supplemental Material.

**FIGURE 6:**
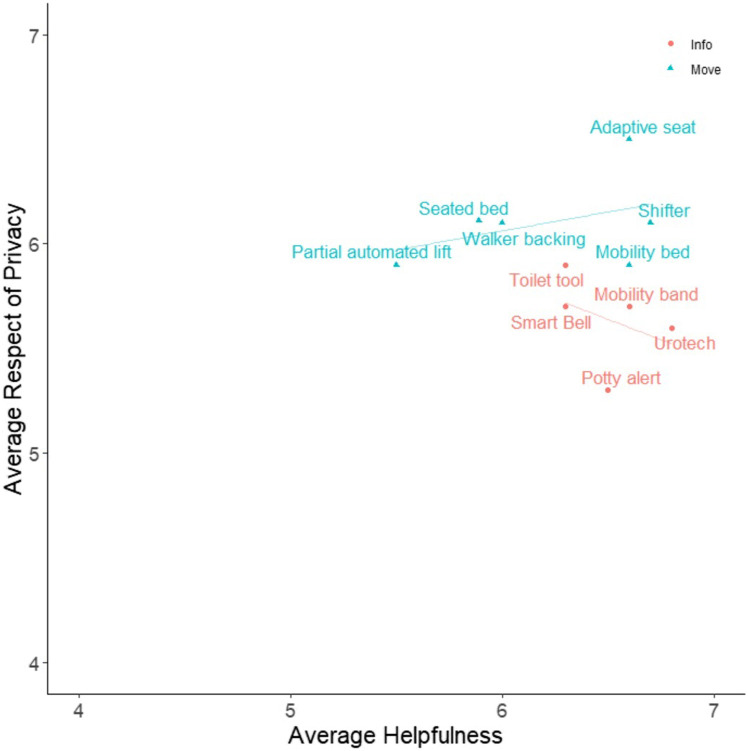
Average Helpfulness vs. Average Respect of Privacy for Participant Designs. Participants reported their own perception of Helpfulness and Respect of Patient Privacy (along with 3 other qualities) on 7 point Likert scales for each participant design (including their own) created during the Co-Creation Session. Averages of these measures for each design are presented in the figure. Designs were qualitatively categorized into one of two groups: tools which provide additional patient information (Info), or tools which aid in moving patients (Move) to assist clinicians in the toileting process. Regression lines of best fit were added to the figures for illustrative purposes, though statistical analyses were not conducted due to the limited sample size. Further information on each design is presented in Table 2 and the Supplemental Material.

Participants’ ratings of each design were examined to gain further insights about nurse perceptions of communication and information (“Info”) and lifting and mobility assistive (“Move”) technologies. Figures 5, 6 show average for how participants rated each design, and can demonstrate perceived tradeoffs between the two types of technology. Regression lines of best fit were added to the figures for illustrative purposes, though statistical analyses were not conducted due to the limited sample size. Figure 5 shows how participants rated designs in terms of Desirability and Ease of Implementation. Info tools, on average, are perceived as easier to implement Move tools. The two designs with the highest Desirability ratings were the Shifter and Urotech, and the tool with the highest Ease of Implementation was Mobility Bands. The two types of designs appear to have different relationships between Desirability and Ease of Implementation, such that Info tools are more desirable as they become more difficult to implement, and Move tools are more desirable as they become easier to implement. Figure 6 shows how participants rated designs in terms of Helpfulness and Respect for Patient Privacy. Here, Move tools were perceived as more respectful of patient privacy on average compared to Info tools. Here, Info tools may be perceived to be less respectful of privacy as they become more helpful. The tool perceived as the most respectful of privacy was the Adaptive Seat, and the tool perceived as most helpful was Urotech. However, due to the limited sample size, statistical analysis was not appropriate and additional study would be necessary to determine if these trends hold true across a larger sample of participants.

Finally, we examined what participants thought about the possibility that robots could be used to totally automate and remove the nurse from the toileting process. None of the participants’ designs featured a robot which fully automated the process. At the end of the session, the concept of a fully automated robotic process was discussed after participants were prompted to think about technology which could be used in 100 years’ time. Only one participant noted a possible benefit, imagining that patients may want a robot because: *“for some people, the privacy [offered by a robot] is better because [toileting] is very vulnerable.”* All other sentiments expressed by participants about the use of a toileting robot were negative. They were concerned that a robot would not be safe because, “*with technology, something can always go wrong… You don’t know the robot will be working properl*y”. Multiple participants were concerned with a robot’s perception and responsiveness to pain: *“A negative for a robot is understanding patients’ pain. Like knowing if moving a patient, if they say ‘stop’ or ‘ow’, you need to know to stop*”. Another participant elaborated on this ability, and stated: “*it would have to be able to tell you what it’s doing as it’s doing it and also be able to—if the patient is like, ‘I need you to stop’ it would just stop. So basically it would have to act like a person*”, because recognizing and quickly responding when someone is in pain is a human capability that nurses use in their work. Another participant stated: “*[a robot] would have to have a certain amount of empathy… It couldn’t be strictly robotic. It would have to be able to communicate and understand that [the toileting process] is uncomfortable*”. This again suggests that nurses can provide therapeutic emotional support when toileting patients which may be challenging for robots to emulate. Participants also expressed concern about the emotional impact on patients if a nurse was removed: “*Even in a hundred years, I feel like most people would rather be cared for by another human rather than being cared for by a robot, like as if you’re just an after-thought.*” In this case, automating the toileting process entirely can send a signal to the patient that the potentially painful and dangerous process of getting up and going to the bathroom is an inconvenience to nurses. This conversation shed more light on the perceived tradeoffs between increased patient privacy and decreased safety and human support for fully autonomous robots in the context of toileting. This again supports the idea that toileting is a valuable component of care that nurses provide, and automation might eliminate the opportunity for support during a physically taxing and potentially emotional part of the patient’s recovery while in the hospital.

## 4 Discussion

Over the course of our investigation, the team discovered that the process of toileting in the hospital was broader than just the mechanical act of bringing a patient to and from a receptacle to void their body at regular intervals; in the hospital, the act of toileting has many physiological, logistical, social, and emotional facets. Patient and nursing staff’s needs surrounding the act of toileting and falls prevention are sometimes aligned and sometimes diverge. On one hand, nurses are experts at recognizing patients’ physiological and emotional status and supporting patients to engage in their recovery by getting out of bed to avoid physical and emotional deterioration. In this way, the act of bringing patients to the bathroom can be beneficial in providing an opportunity for frequent patient-nurse interactions and assessment, encouragement to get up and moving to promote healing, and the opportunity to maintain dignity by performing necessary and normal human functions behind a closed door. However, if the nurse is overloaded with work or is assigned too many patients, the nursing time demand for toileting can become overwhelming or frustrating, and can lead to long patient wait times, a decision to lift a patient alone when it is ergonomically unsafe, leaving the patient alone in the bathroom to complete other work, or the decision to force a patient to void in the bed using a bedpan or other device. For patients, the urgency in bathroom need, or the desire to maintain privacy and autonomy in an embarrassing situation, coupled with the desire to not inconvenience staff or avoid being forced to use a dehumanizing method for toileting can lead to the decision to not comply and take risks.

While hospitals already use tools to estimate risk, alert nursing staff when a patient is getting up on their own, and lift and move patients, there are many environmental, contextual, and human factors that affect the utility of any technology in this context. As noted by our participants, a patient’s susceptibility of falling can fluctuate over time as their conditions change, and therefore the nurse must use expert judgment to reassess the patient in the moment. If all patients are estimated to be a falls risk, then the scores provide no utility when prioritizing resources for patients. AI technology might prove useful if it can provide a better stratification of patients and estimate toileting needs and fall risks in the moment. However, such an algorithm would need access to pertinent data points in real-time and a means of conveying this estimate to nurses in a manner that enables them to help.

Physically lifting a patient and helping them walk can put nursing staff in dangerous and awkward postures that put them at risk of occupational injury, but waiting for an additional staff person to help or using lifting and mobility assistive tools present their own challenges. Cramped hospital rooms and bathrooms coupled with the other bulky equipment needed by patients make navigation challenging, and mobility assistive tools are often not available or easily accessible when needed. Crucially, a patient can experience pain in the process of being lifted or moving, so the nurse needs to be able to stop at a moments’ notice. All of these constraints pose significant challenges for a physical robot which could operate in this environment. A mobile robot base would be challenged in navigating the environment, and could cause additional challenges if it collided with equipment, other people, or if the unsteady patient caused the robot itself to fall while it assisted them. Furthermore, the use of an autonomous robot could make patients feel unsafe, especially in moments that are very painful. Totally removing the nurse could make the patient feel like they are an inconvenience when they really need support, which could undermine their trust or willingness to engage further with the nurse.

When considering the design of AI and robotic technology, functionality will need to be balanced against usefulness and potential consequences. We found that participants in the Co-Creation session imagined ideal tools to include elements which supported communication or more holistic information, which AI tools could be designed to assist with, as well as mobility assistive applications, which may incorporate robotics. Instead of a fully autonomous robot, robotic elements could be added to existing fixtures within the hospital, such as beds, walkers, and toilets. This would allow them to provide support by being universally available when needed, while adding minimal mass to the existing environment. Participant ratings of tools designed in the Co-Creation session offer additional insights on the potential tradeoffs to be considered for AI technology. AI tools which are desirable and helpful might also be more challenging to implement and may ask patients to relinquish some privacy as their status and “fullness” is tracked. Info tools which include predictive power and integration with existing software and hardware will be harder to implement, but things which are highly integrated may provide better utility. Mobility assistive devices, on the other hand, may become more desirable and helpful as they become easier to implement and more respectful of patient privacy. This may indicate that robotic devices which do less to change the process or add bulk to the environment may be seen as less cumbersome and more respectful to patients. Ultimately, designers creating any technology for healthcare contexts need to pay careful attention to human needs in order to balance the tool capabilities against the challenges they introduce.

### 4.1 Our design philosophy for fall preventative AI and robotic technology

Taken together, these findings revealed to us that getting patients out of bed to perform the normal and necessary human function of toileting created the opportunity for a valuable patient-nurse touchpoint, rather than a nuisance which should be automated. However, the process of getting a patient up and to the bathroom and back can and should be ameliorated by addressing major pain points, including nursing time demands and interruptions, the poor ergonomics and risk of occupational injury in lifting and moving patients, and the patient’s physical and emotional comfort through the process. Technologies like AI and robotics can be designed to enhance this process, but careful attention must be paid to contextual and human factors during the design process to reduce the risk of lack of adoption and unintended consequences. As our team is currently engaging in additional human-centered research and design surrounding these types of tools, we have intentionally avoided a thorough survey of current work on this topic to avoid the introduction of bias in our design process. Individuals interested in learning more may find additional insight in recent systematic reviews (Ren and Peng, 2019; Usmani et al., 2021).

Considering our findings during this investigation, our team adopted a design philosophy which included the following beliefs:

1. Patients should use the toilet in the bathroom whenever they are physically capable with or without assistance, instead of a bedside commode, bedpan, chucks, or any other device. When this is not possible due to extreme weakness or acute injury, returning to the bathroom should be a care goal. We believe that toileting in the bathroom is not only more sanitary and comfortable, it preserves patient dignity and encourages engagement in maintaining or recovering independence. We therefore believe technologies should be designed to assist with toileting in the bathroom rather than alternative means, regardless of the potential convenience.
2. The patient-nurse interaction during toileting is a valuable touchpoint, which provides the opportunity for assessment and therapeutic support from caregivers. Therefore, we believe that the interaction should be preserved (in part or in whole), even as robotic systems advance to the point where full automation is an option. If robots are developed and implemented to assist in the context of patient toileting, we believe designers and healthcare organizations should take steps to ensure that this valuable patient-nurse touchpoint isn’t entirely lost and to pay careful attention to the tradeoffs and potential for unintended consequences when altering this interaction.

Human-centered design is necessary to recognize where and how AI and robotic tools can provide the greatest value in healthcare contexts. A commitment to finding human needs at the beginning of this process helps to identify major design issues before building systems that are very costly and difficult to change. Initial design concepts developed by engineering teams (e.g., our initial idea that a robot could bring patients to the bathroom instead of nurses to reduce falls) should be thoroughly examined prior to commitment to recognize potential for design failure and other unintended consequences, as well as to uncover new insights that can inform better human-centered products. Research presented in this article represents a “deep dive” into clinician and patient needs surrounding “high-tech” toileting tools, and is the first step in a planned series of studies. Future work will build upon this research by surveying a larger pool of clinicians to verify the generalizability of our findings, and by conducting additional research and design iterations to develop tools for a safer and more comfortable toileting process for patients and clinicians alike.

## 5 Limitations

This work is limited in a number of ways. Our participants were convenience and snowball sampled, and all participants lived within the state of New York. While our sample size is typical of this type of qualitative design research due to the large volume of qualitative data which must be analyzed, the total number of participants is small, and our results should be interpreted with appropriate caution. Further research is necessary to confirm the generalizability of our findings. Additionally, while we wanted to get the patients’ perspective, it was impractical to recruit patients for our study to meet our timeline, therefore we used the vignettes to prime participants to put themselves in the position of being a patient. In future work, individuals who had experienced hospitalization and needed assistance with toileting, or individuals who had experienced a fall while hospitalized would add valuable insight.

## Data Availability

The original contributions presented in the study are included in the article/Supplemental Material, further inquiries can be directed to the corresponding author.

## References

[B1] Anzaldua A. Halpern J. (2021). Can clinical empathy survive? Distress, burnout, and malignant duty in the age of covid-19. Hastings Cent. Rep. 51 (1), 22–27. 10.1002/HAST.1216 PMC8013970 33630324

[B2] Capo‐Lugo C. E. Young D. L. Farley H. Aquino C. McLaughlin K. Colantuoni E. (2023). Revealing the tension: the relationship between high fall risk categorization and low patient mobility. J. Am. Geriatrics Soc. 71 (5), 1536–1546. 10.1111/jgs.18221 PMC10175187 36637798

[B3] Childers C. P. Maggard-Gibbons M. (2018). Estimation of the acquisition and operating costs for robotic surgery. JAMA 320 (8), 835. 10.1001/jama.2018.9219 30167686 PMC6142989

[B4] Christoforou E. G. Avgousti S. Ramdani N. Novales C. Panayides A. S. (2020). The upcoming role for nursing and assistive robotics: opportunities and challenges ahead. Front. Digital Health 2, 585656. 10.3389/fdgth.2020.585656 PMC8521866 34713058

[B5] Downe‐Wamboldt B. (1992). Content analysis: method, applications, and issues. Health Care Women Int. 13 (3), 313–321. 10.1080/07399339209516006 1399871

[B6] Dykes P. C. Curtin-Bowen M. Lipsitz S. Franz C. Adelman J. Adkison L. (2023). Cost of inpatient falls and cost-benefit analysis of implementation of an evidence-based fall prevention program. JAMA Health Forum 4 (1), e225125. 10.1001/JAMAHEALTHFORUM.2022.5125 36662505 PMC9860521

[B7] Florence C. S. Bergen G. Atherly A. Burns E. Stevens J. Drake C. (2018). Medical costs of fatal and nonfatal falls in older adults. J. Am. Geriatr. Soc. 66, 693–698. 10.1111/jgs.15304 29512120 PMC6089380

[B8] Frijda N. (1986). The emotions. Cambridge: Cambridge University Press.

[B9] Goldsborough K. A. Goldsborough K. Samuels J. (2019). Implementation of safe patient toileting to decrease patient falls implementation of safe patient toileting to decrease patient falls on medical-surgical unit on medical-surgical unit implementation of safe patient toileting to decrease patient falls on medical-surgical unit. Available at: https://scholars.unh.edu/scholarly_projects/30.

[B10] Hignett S. Wolf L. (2016). Reducing inpatient falls: human Factors & Ergonomics offers a novel solution by designing safety from the patients’ perspective. Int. J. Nurs. Stud. 59, A1–A3. 10.1016/J.IJNURSTU.2016.02.007 26924377

[B11] Hitcho E. B. Krauss M. J. Birge S. Dunagan W. C. Fischer I. Johnson S. (2004). Characteristics and circumstances of falls in a hospital setting: a prospective analysis. J. General Intern. Med. 19 (7), 732–739. 10.1111/J.1525-1497.2004.30387.X PMC1492485 15209586

[B12] Jacobs A. (2021). ‘Nursing is in crisis’: staff shortages put patients at risk - the New York times. New York, NY: The New York Times. October 20 Available at: https://www.nytimes.com/2021/08/21/health/covid-nursing-shortage-delta.html.

[B13] Khachiyants N. Trinkle D. Son S. J. Kim K. Y. (2011). Sundown syndrome in persons with dementia: an update. Psychiatry Investig. 8 (4), 275. 10.4306/PI.2011.8.4.275 PMC3246134 22216036

[B14] Li L. Foo M. J. Chen J. Tan K. Y. Cai J. Swaminathan R. (2023). Mobile Robotic Balance Assistant (MRBA): a gait assistive and fall intervention robot for daily living. J. NeuroEngineering Rehabilitation 20 (1), 29. 10.1186/s12984-023-01149-0 PMC9979429 36859286

[B15] Mansouri N. Goher K. Hosseini S. E. (2017). Ethical framework of assistive devices: review and reflection. Robotics Biomimetics 4 (1), 19. 10.1186/s40638-017-0074-2 29201602 PMC5688189

[B16] Marschollek M. Gövercin M. Rust S. Gietzelt M. Schulze M. Wolf K.-H. (2012). Mining geriatric assessment data for in-patient fall prediction models and high-risk subgroups. BMC Med. Inf. Decis. Mak. 12 (1), 19. 10.1186/1472-6947-12-19 PMC3314576 22417403

[B17] McFadden M. (2018). Robots to the rescue: helping monitor patients at risk of falls. confusion Available at: https://www.wndu.com/content/news/Robots-to-the-rescue-Helping-monitor-patients-at-risk-of-falls-confusion-493777071.html.

[B18] McHugh M. L. (2012). Interrater reliability: the kappa statistic. Biochem. Medica 22 (3), 276–282. 10.11613/bm.2012.031 PMC3900052 23092060

[B19] Namba T. Yamada Y. (2018). Risks of deep reinforcement learning applied to fall prevention assist by autonomous mobile robots in the hospital. Big Data Cognitive Comput. 2 (2), 13. 10.3390/bdcc2020013

[B20] Novin R. S. Yazdani A. Merryweather A. Hermans T. (2021). “Risk-aware decision making for service robots to minimize risk of patient falls in hospitals,” in 2021 IEEE International Conference on Robotics and Automation (ICRA), Xian, China, May 30 to June 5, 2021, 3299–3305. 10.1109/ICRA48506.2021.9560871

[B21] Oh-Park M. Doan T. Dohle C. Vermiglio-Kohn V. Abdou A. (2021). Technology utilization in fall prevention. Am. J. Phys. Med. Rehabilitation 100 (1), 92–99. 10.1097/PHM.0000000000001554 32740053

[B22] OSHA (2023). Healthcare - safe patient handling | occupational safety and health administration. Available from: https://www.osha.gov/healthcare/safe-patient-handling (Accessed September 4, 2023).

[B23] Owen B. D. Welden N. Kane J. (1999). What are we teaching about lifting and transferring patients? Res. Nurs. health 22 (1), 3–13. 10.1002/(sici)1098-240x(199902)22:1<3::aid-nur2>3.0.co;2-s 9928959

[B24] Pepito J. A. Locsin R. (2019). Can nurses remain relevant in a technologically advanced future? Int. J. Nurs. Sci. 6 (1), 106–110. 10.1016/J.IJNSS.2018.09.013 31406875 PMC6608671

[B25] Ren L. Peng Y. (2019). Research of fall detection and fall prevention technologies: a systematic review. IEEE Access 7, 77702–77722. 10.1109/access.2019.2922708

[B26] Saadatzi M. N. Logsdon M. C. Abubakar S. Das S. Jankoski P. Mitchell H. (2020). Acceptability of using a robotic nursing assistant in health care environments: experimental pilot study. J. Med. Internet Res. 22 (11), e17509. 10.2196/17509 33180024 PMC7691087

[B27] Schoenfisch A. L. Kucera K. L. Lipscomb H. J. McIlvaine J. Becherer L. James T. (2019). Use of assistive devices to lift, transfer, and reposition hospital patients. Nurs. Res. 68 (1), 3–12. 10.1097/nnr.0000000000000325 30540690 PMC13101446

[B28] Staggs V. S. Mion L. C. Shorr R. I. (2014). Assisted and unassisted falls: different events, different outcomes, different implications for quality of hospital care. Jt. Comm. J. Qual. Patient Saf. 40 (8), 358–364. 10.1016/S1553-7250(14)40047-3 25208441 PMC4276137

[B29] Tang R. Poklar M. Domke H. Moore S. Kapellusch J. Garg A. (2017). Sit-to-stand lift: effects of lifted height on weight borne and upper extremity strength requirements. Res. Nurs. Health 40 (1), 9–14. 10.1002/NUR.21754 27686534

[B30] Tay J. L. Xie H. T. (2023). Novel interventions significantly reduce falls with fractures: a meta-analysis and systematic review. Geriatr. Nurs. 52, 181–190. 10.1016/j.gerinurse.2023.06.004 37390566

[B31] Usmani S. Saboor A. Haris M. Khan M. A. Park H. (2021). Latest research trends in fall detection and prevention using machine learning: a systematic review. Sensors 21 (15), 5134. 10.3390/s21155134 34372371 PMC8347190

[B32] Wilkinson J. (2015). *The strong robot with the gentle touch*. Riken. Available at: https://www.riken.jp/en/news_pubs/research_news/pr/2015/20150223_2/.

[B33] Zoom (2023). One platform to connect | Zoom. Available from: https://zoom.us/ (Accessed September 15, 2023).

