# Supplementary material for: Towards human-centered AI and robotics to reduce hospital falls: finding opportunities to enhance patient-nurse interactions during toileting
Source: Front Robot AI. 2024 Jan 31;11:1295679. doi: 10.3389/frobt.2024.1295679 (PMC10865095; doi:10.3389/frobt.2024.1295679)

Supplementary Material

# Supplementary Data

## Interview Questions

1. Demographics:
   1. What is your self-described gender?
   2. Can you describe your job role?
   3. How long have you worked in this role?
   4. How many years in total have you worked in healthcare?
2. From your experience, what has the primary cause of patient falls been?
3. In the process of getting up and out of bed to go to the bathroom, which movements/activities are more challenging for patients?
4. Can you walk through the process of a hospitalized patient going to the bathroom?
5. When assisting a patient to the bathroom, what are some things you struggle with most?
6. How does your hospital prevent falls currently?
7. Is it more valuable for technology to bring the patient to the bathroom or help them in the bathroom?
8. Do you think patients will have an increase in their privacy with technology in the bathroom rather than a human?
9. What kind of patients would this kind of technology be useful for?
10. What patients would you be least likely to use this for?
11. What kind of space concerns might you have for some type of technology?
12. How would you benefit from some type of technology aiding in bathroom procedures and falls? How would it affect your responsibilities?

## Focus Group Questions and Vignette Description

1. Demographics:
   1. What is your job role?
   2. How long have you been in this role?
   3. How long have you been working in healthcare?
2. General questions before showing the videos
   1. From your experience, what are some of the primary reasons patients don’t ask for help when they need to use the bathroom?
   2. When you get a bathroom call, what is the most challenging part once you get to the room to help them?
3. Questions after each vignette:
   1. How do you think the patient feels?
   2. What would you want to change about this situation if it was you?
4. Wrap-Up Questions
   1. Imagine a device, like a robot or AI is created, how could it solve the issues that we have discussed?
   2. If it was designed to do [whatever was discussed], what would we need to look out for? What could go wrong in design, or when using the tool?
   3. Would a person feel more or less comfortable, or have a different sense of privacy around a robot/AI than a nurse/clinician?
   4. Is there anything else that’s top of mind for you?

## Focus Group Vignette Descriptions

Vignette 1:

The scene opens with a patient sitting in a hospital bed. She has an urgent need to use the bathroom, and states that she will “not bother with” the call bell, and gets out of bed on her own. When she tries to get to her feet, the patient experiences dizziness and falls on the ground. A few moments later, a nurse arrives to find the patient on the floor. The nurse assesses the patient, and calls for a “rapid response”, in which others come to assist with an emergency situation. When someone responds, she asks for a stretcher because the patient hit her head when she fell getting out of bed.


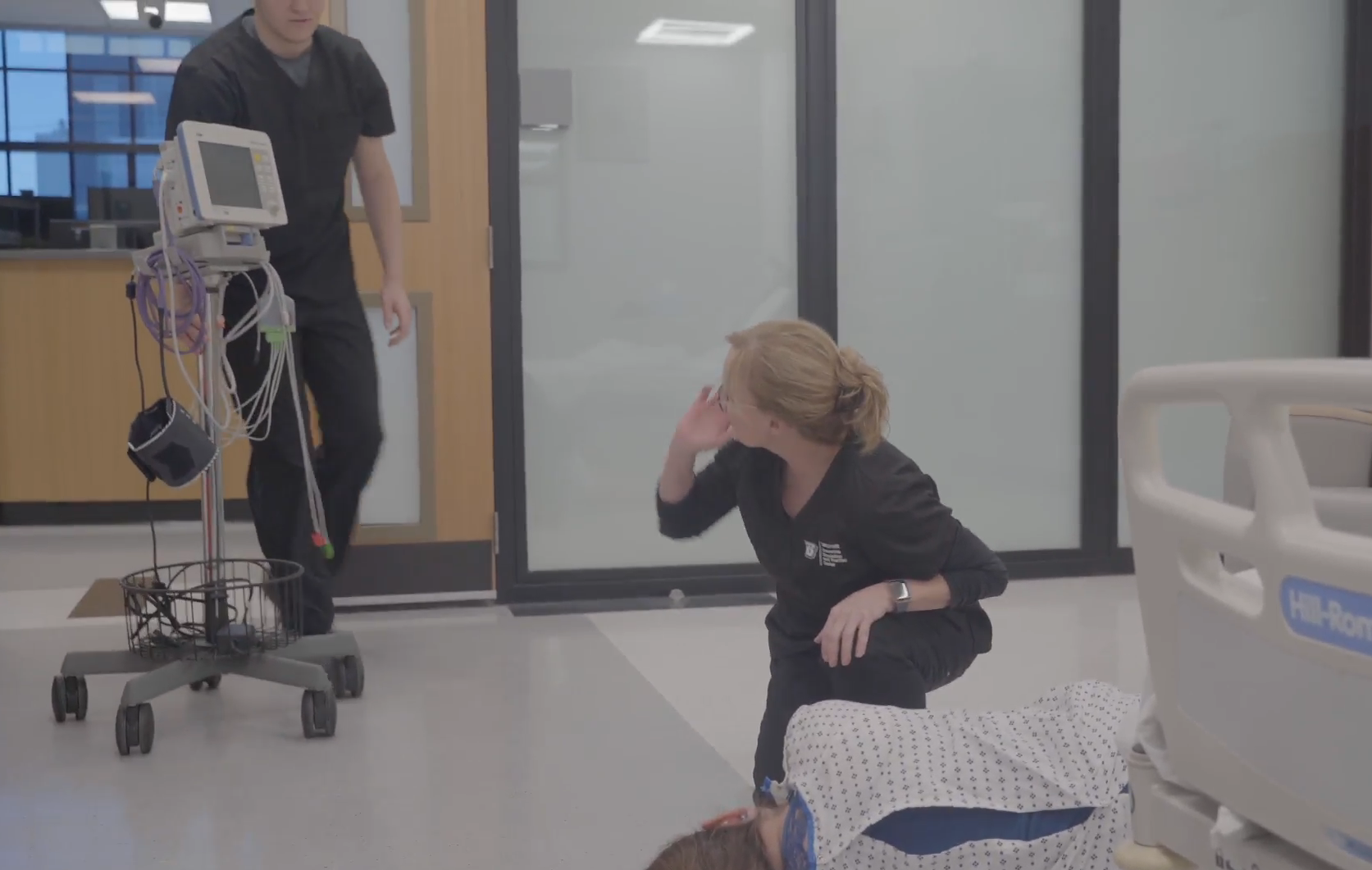


**Supplementary Figure 1**: Still image from Vignette 1 video. The patient has fallen, and her nurse called for a rapid response.

Vignette 2:

The scene opens with a younger male patient wearing a leg brace who says he needs to use the bathroom, but he doesn’t see anyone, as he is talking to his friend. The friend tells the patient to hit the call bell, but the patient says he doesn’t want to inconvenience busy staff. When he does finally hit the call bell, he is annoyed that no one responds immediately. As he waits, he becomes increasingly frustrated by the fact that they need assistance due to the injury. Once the nurse shows up, the patient says “finally” and acts annoyed with the nurse.


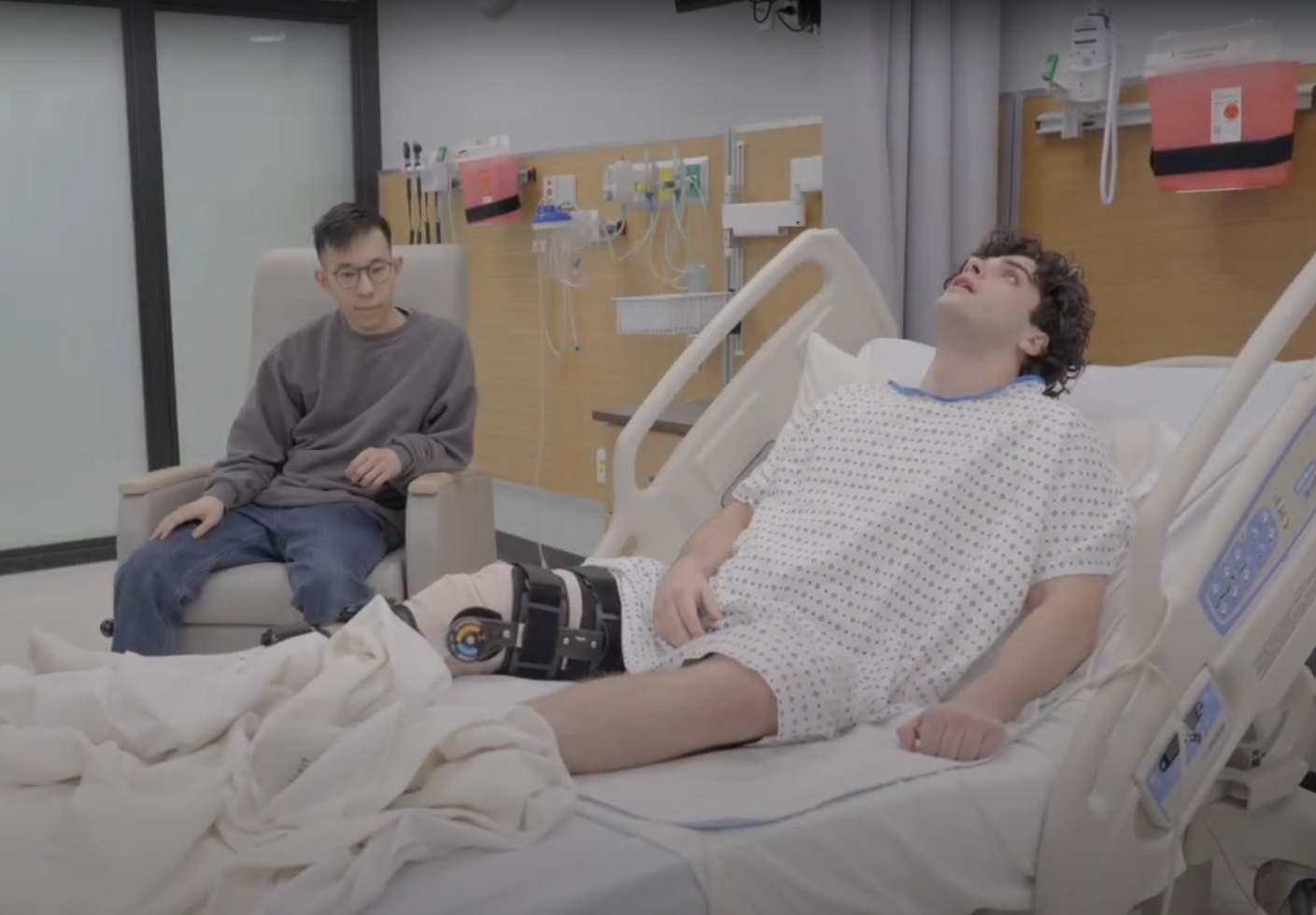


**Supplementary Figure 2**: Still image from Vignette 2 video. The patient has an urgent need to use the bathroom, but did not want to inconvenience his nurse.

Vignette 3:

The scene opens with an elderly female patient (a simulation dummy) in bed. Two younger male nurses come into her room and immediately say, “Hi sweatheart! We heard you called for the bathroom”. They have a leisurely pace as she asks them to hurry up. They tell her that she has too much going on, and that she will need to use the bedpan. As they position her to use the bedpan, someone from housekeeping comes to the door with full view of the patient. The nurses say it’s not a good time, and the person leaves. After the patient finishes, the nurses rotate her to her side so they can wipe her, and two of the patient’s grandchildren show up and walk right into the room. The patient is silent, and nurses ask them to come back later, and finish up with the patient.


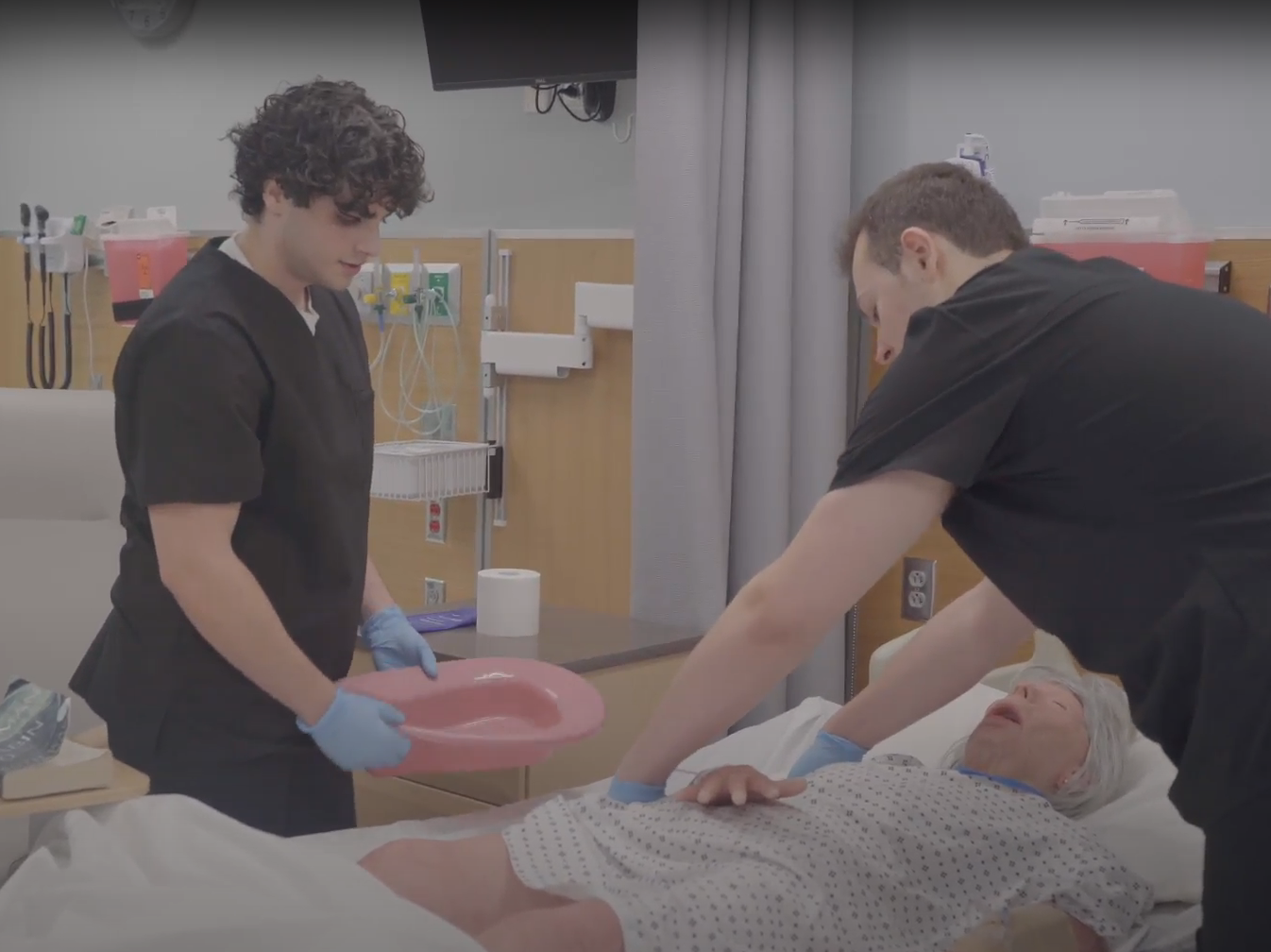


**Supplementary Figure 3**: Still image from Vignette 3 video. Two younger male nurses place a bedpan under an elderly female patient instead of taking her to the bathroom.

## Co-Creation Session Outline

1. **PART ONE (35 minutes)**: **Get more clarification on general ideas from perspective of clinicians**
   1. **Activity 1:** **Rapid ideation on post-it notes (12 minutes)**
      1. Every idea was written on an individual post-it note. For two minutes per prompt, participants came up with as many ideas as they could think of for the following prompts: What tools are used or could be used to help…
         1. Identify that a patient needs to go to the bathroom?
         2. Understand urgency in bathroom needs?
         3. Physically assist patients out of bed, to the bathroom and back, back to bed?
         4. Ensure patient comfort in the process?
         5. Ensure patient compliance with protocol?
      2. Participants were given different colored post-its, and instructed to color-code their ideas which relate to:
         1. Social concepts = Green
         2. Human body concepts = Red
         3. Protocols or tools = Blue
   2. **Activity 2:** Post-it Organization (25 minutes)
      1. Participants organized post-its on a How-Now-Wow chart, where:
         1. How: innovate, but not desirable
         2. Now: not innovation, desirable or not, typically things currently in use
         3. Wow: innovative and desirable
      2. After ~20 minutes, each group picked a member to explain their chart and reasoning
2. **PART TWO (30 minutes): Design your tool/device**
   1. Participants create their own ideal tool using craft supplies provided, including sample print outs of a human body diagram, hospital room layout, phone screen, and EHR screen.
   2. Participants encouraged to consider what their tool does, who it is used by, how many people it is used by, and what task(s) would it would fulfill. Participants also informed that they will share it with the group.
3. **PART THREE (15 minutes): Share out and anonymously rate designs**
   1. Participants gave their work a title, and described it to the group, including why it was useful and what it accomplished.
   2. All participants anonymously rated each design (including their own) on a Likert from 1 – 7 for the following qualities:
      1. Helpful
      2. Respectful of privacy
      3. Ease of implementation
      4. Desirable
      5. Ease of use
4. **PART FOUR (15 minutes): Technology in 100 years**
   1. Prompt: Think about the world 100 years from now, with technology such as robots, automation, and AI integrated into our society. Consider the following:
      1. What is one type of innovation that you can imagine in a design to assist with toileting that is not already in use?
      2. How will this technology affect the patient?
      3. Ignoring present day limitations, what would be the ideal method of patients going to the bathroom at the hospital?
5. **PART FIVE (10 minutes): Open ended discussion**
   1. Is there anything else you wanted to add based on these discussions and activities today?

## Qualitative Content Analysis Results

**Supplementary Table 1**: Initial codes. Definitions and counts are presented with percent agreement and Cohen’s Kappa to estimate inter-rater reliability.

| Code | Definition | Count | Percent agreement | Cohen's Kappa |
| --- | --- | --- | --- | --- |
| Communication | active, communicating info between at least 2 parties (pt and nurse), method by which knowledge/info is exchange; also displays of visual or auditory information | 30 | 0.71 | 0.66 |
| Patient knowledge | Holistic patient knowledge, medications, fall risk, bathroom tendencies, medical history, needs, in the moment if they will need to use the bathroom; including intuition -- | 28 | 0.75 | 0.67 |
| Access to device / tool | if the tool is available to use when needed; storage, boundaries of physical object/floor space requirements, abundance within hospital | 20 | 0.79 | 0.74 |
| Pain / injury / body mechanics | a person being in pain or getting injured, especially related to movement of the body or medical conditions/IVs etc. | 20 | 0.78 | 0.66 |
| Empathy | Take into account the patient feelings and emotions (recognizing, paying attention to, understanding), and clinician emotions, respect, privacy | 14 | 0.86 | 0.69 |
| Timelines | Anything related to things related to time, e.g., knowing that patient just ate lunch and will need to go soon; or not having enough time or feeling rushed | 8 | 0.92 | 0.71 |
| Valence | Is the statement positive (+1), negative (-1), or neutral (0) |  | 0.73 | 0.69 |

All of the Kappa values fell within the range of 0.61 to 0.80, indicating that raters had substantial agreement in applying codes to the data. The team worked to iteratively refine codes by applying these to data, checking agreement and discussing as a team. The team split up the remaining data such that each data source (e.g., an individual transcript) was coded by 2 researchers. The only exceptions were post-its within the How, Now, Wow activity, and the second focus group, which were coded individually.

The codes privacy and patient autonomy were added to the code book as they emerged as important topics during coding and conversation. Over the course of iteratively refining codes, the team recognized that two codes (body and availability) occurred much more frequently than others. Body was then broken down into two codes: body: involving body movements and body mechanics, and pain: anything related to injury and experiences of physical pain. Availability was split into access, regarding access and availability of tools, equipment, or personnel, and process, to indicate steps in the toileting process as it occurs in current practice, as stated by participants. “Empathy” was also updated to “emotions”, which encompassed more concepts. In the end, 10 codes were established and applied to all data (see Table 2).

**Supplementary Table 2**: Codes, definitions, representative quotes, counts, average valence, and percent agreement. Codes are presented from most to least frequently occurring.

| **Code** | **Definition** | **Representative Quote** | **Count** | **Average Valence** | **Percent agreement** |
| --- | --- | --- | --- | --- | --- |
| Process | Involving all existing protocols and tools that are used in the hospital, processes that nurses go through when taking patients to the bathroom | “High [falls] risk patients get a portable commode in the room and 24/7 supervision when going to the bathroom” (Interview 1) | 136 | 0.15 | 0.76 |
| Body | Movement of the body (patient or staff), or medical conditions. | “[Some patients have] orthostatic hypotension when sitting up or standing too fast, which causes blood pressure to drop and causes dizziness and lightheadedness” (Interview 2) | 134 | 0.10 | 0.83 |
| Patient knowledge | Any knowledge or intuition of the patient such as medications, fall risk, bathroom tendencies, medical history, or needs | “Are they Lightheaded? Are they dizzy? Do they have chest pain? Do you feel weak? Do you feel they're gonna pass out? Or do you feel like you're doing okay? And I'll continuously assess them.” (Interview 5) | 130 | 0.23 | 0.85 |
| Availability | If tools/resources are available to use when needed, such as personnel, floor space, or equipment | “Accessibility [of devices] is more important than functionality” (Co-Creation) | 120 | 0.18 | 0.83 |
| Emotions | Anything related to varying patient emotions, mental health, depression, lack of motivation to get up, and displays of empathy/understanding from nursing staff | “We don’t want to make the patient feel like they’re rotting [by sitting alone in bed for too long]” (Focus Group 1) | 83 | -0.19 | 0.90 |
| Communication | Communicating info between at least 2 parties (e.g., a patient and nurse), methods by which knowledge/info is exchanged, and any displays of visual or auditory information | “Communicating with the patient what you are doing before it happens” (Co-Creation, “Now” section of How-Now-Wow) | 81 | 0.42 | 0.90 |
| Autonomy | Ability for patients to carry out tasks independently, with little to no assistance required from nurses and staff | “Elder patients have pride and don’t want to give up independence. They don’t want to admit they are incapable and can’t do things alone or without assistance” (Focus Group) | 71 | 0.11 | 0.90 |
| Privacy | Taking into account a patient's privacy and security; maintaining patient's dignity as a human and person; information and data security | “Young people with bad injuries may need more privacy and may have a preference for a robot [to assist in toileting]” (Interview 5) | 42 | 0.01 | 0.95 |
| Time | Anything related to time, like knowing that patient just ate lunch and will need to go soon, or not having enough time or feeling rushed | “Lack of staff makes it difficult since it can be a 20-30 minute process from bed to bathroom, to waiting, then back to bed, and there are other tasks that need to get done” (Interview 2) | 37 | 0.05 | 0.96 |
| Pain | A person (patient or staff) being in pain, getting injured, or at high risk of injury | “[my design] reduces staff movement injuries like back problems, etc.” (Co-creation, “The Shifter”) | 23 | -0.50 | 0.96 |
| Valence | Is the statement positive (+1), negative (-1), or neutral (0) |  |  |  | 0.71 |

## Co-Creation Designs

**
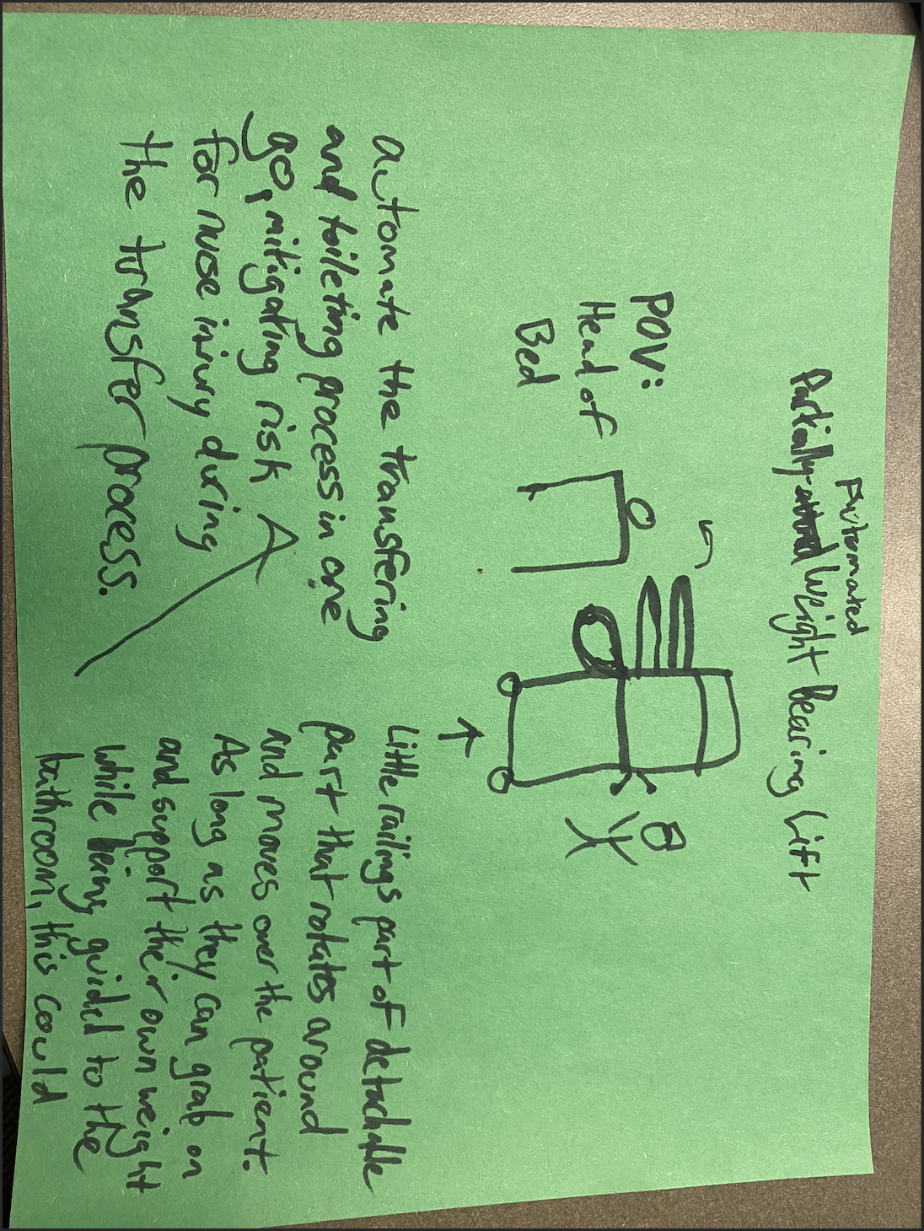
**

Supplemental Figure 1: Design #1: Partial Automated Weight Bearing Lift

The first design was titled the “Partial Automated Weight Bearing Lift”. This design was created to be a combination between a walker and Hoyer lift, and support the weight of a patient. It would move, rotate, and retract, allowing the patient to easily sit on the bed. It would also detach and help take the patient all of the way to the bathroom as long as the patient is able to support their own weight. This would help alleviate pressure and stress from the nurse, since moving the patient to the correct position can potentially hurt the nurse.

Supplemental Table 1: Design #1: Partial Automated Weight Bearing Lift Average Ratings

**
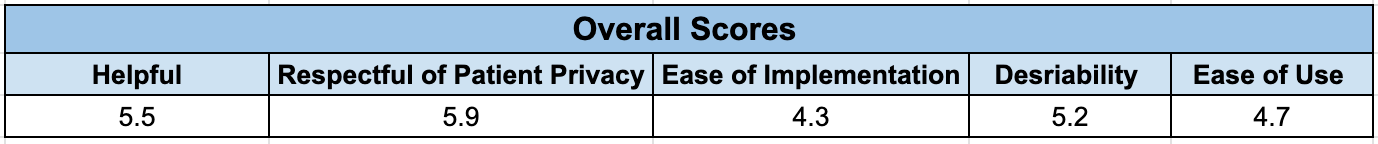
**

**
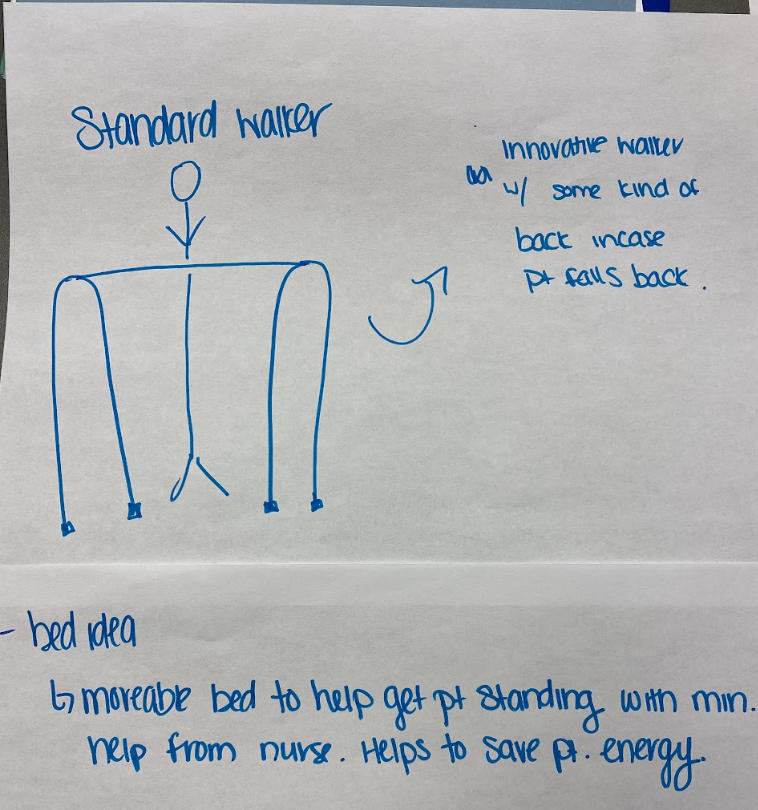
  
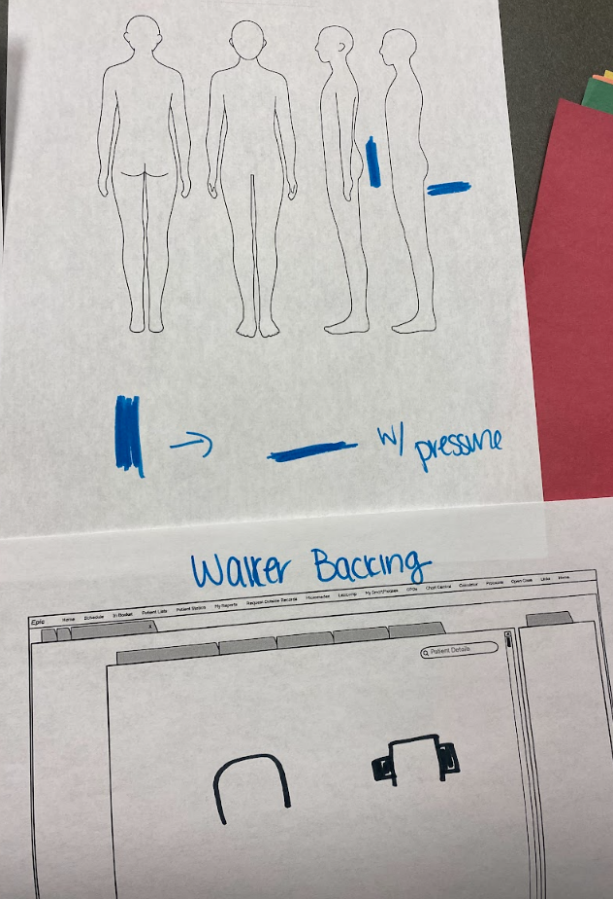
**

Supplemental Figure 2: Design #2: Walker Backing

The second design was titled “Walker Backing”. It would give the patients a support to either sit or lean on and is based on a standard walker. Sturdy flaps would rotate in from the sides of the device and come underneath the patient, which would provide support in case they fall backwards.

Supplemental Table 2: Design #2: Walker Backing Average Ratings


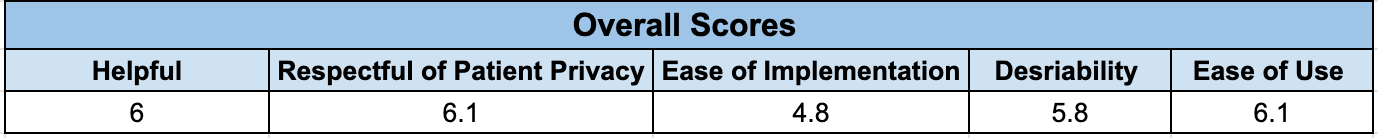


**
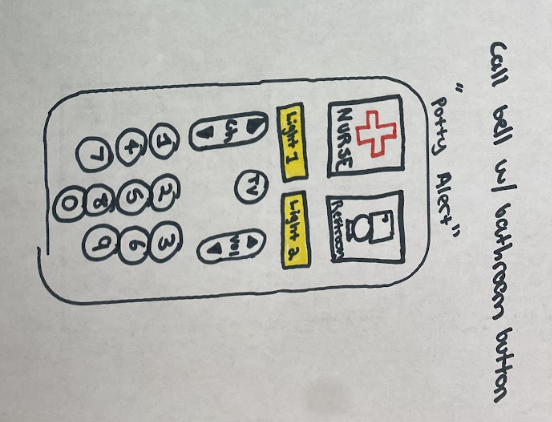
**

Supplemental Figure 3: Design #3: Potty Alert

The third design was titled “Potty Alert”. This design is a call bell with multiple added functions. It has a specific button for nurses as well as having the other buttons for adjusting the TV and room lights. The nurses’ station would hear different chimes for bathroom requests, so they would understand what the patient needs and increase sense of urgency for the nurses.

Supplemental Table 3: Design #3: Potty Alert Average Ratings


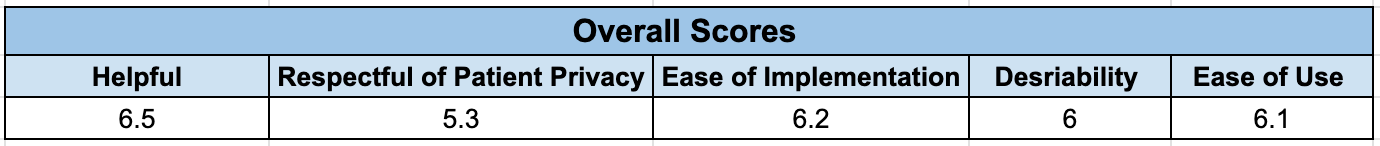


**
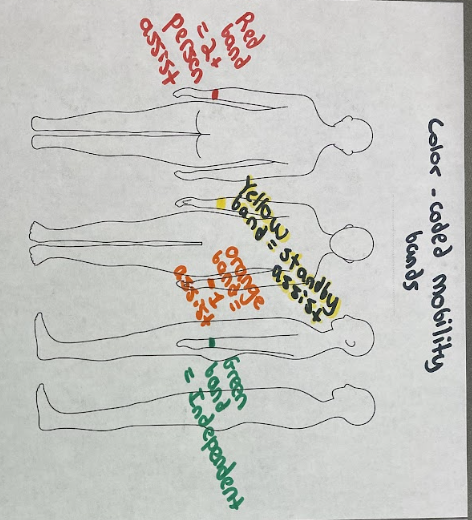
**

Supplemental Figure 4: Design #4: Mobility Bands

The fourth design was titled “Mobility Bands”. It was a simple communication tool that allows nurses to easily identify how many nurses need to assist the patient when they are moved. The red band indicates that the patient needs two or more nurses to assist them, the orange represents one nurse, the yellow represents the need for a standby nurse, and the green band indicates that the patient is independent enough to walk alone.

Supplemental Table 4: Design #4: Mobility Bands Average Ratings


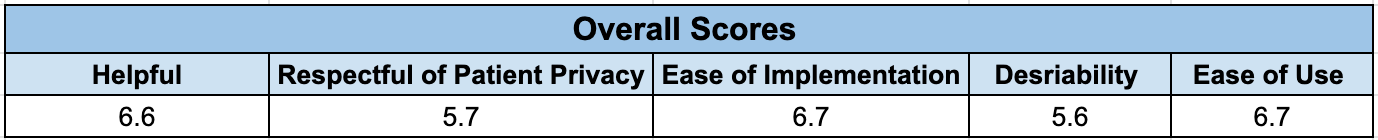


**
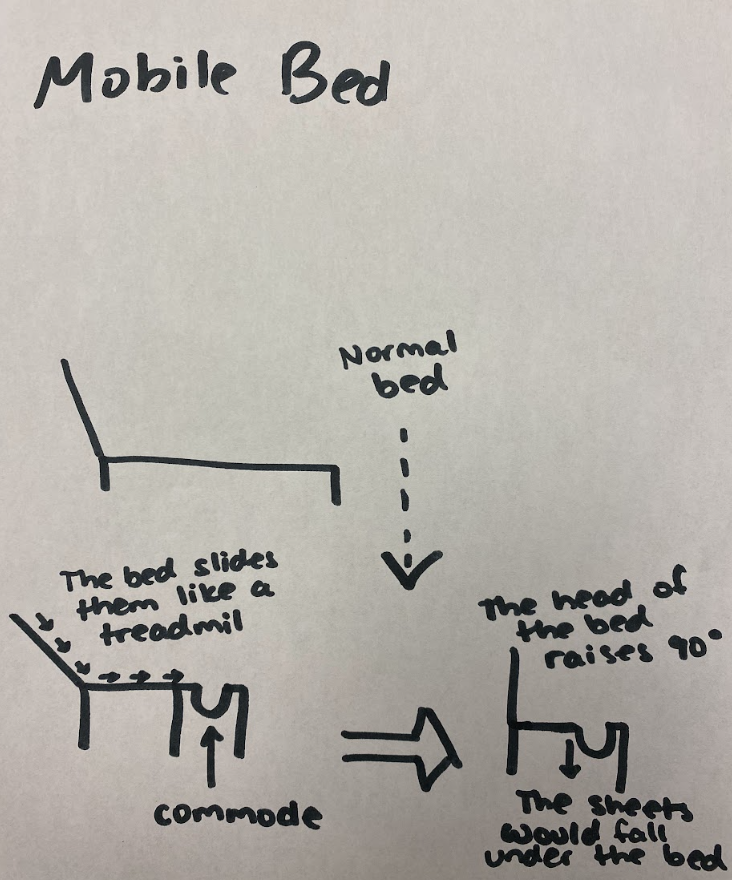
**

Supplemental Figure 5: Design #5: Mobile Bed

The fifth design was titled “Mobile Bed”. It is similar to a normal bed but has a head that turns 90 degrees. Nurses would be able to remove the lower half of the bed, converting it into a commode. The sheets of the bed would fall into slits between the bed and commode, and the entire process would be overseen by a nurse. There would also be a treadmill feature that would slide the patient down to the edge of the bed, which would allow the patient to be easily moved.

Supplemental Table 5: Design #5: Mobile Bed Average Ratings

**
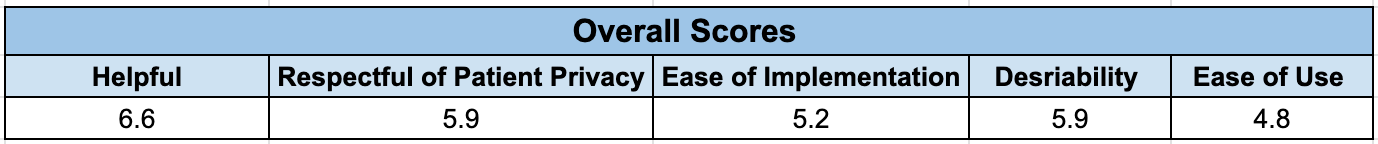
**

**
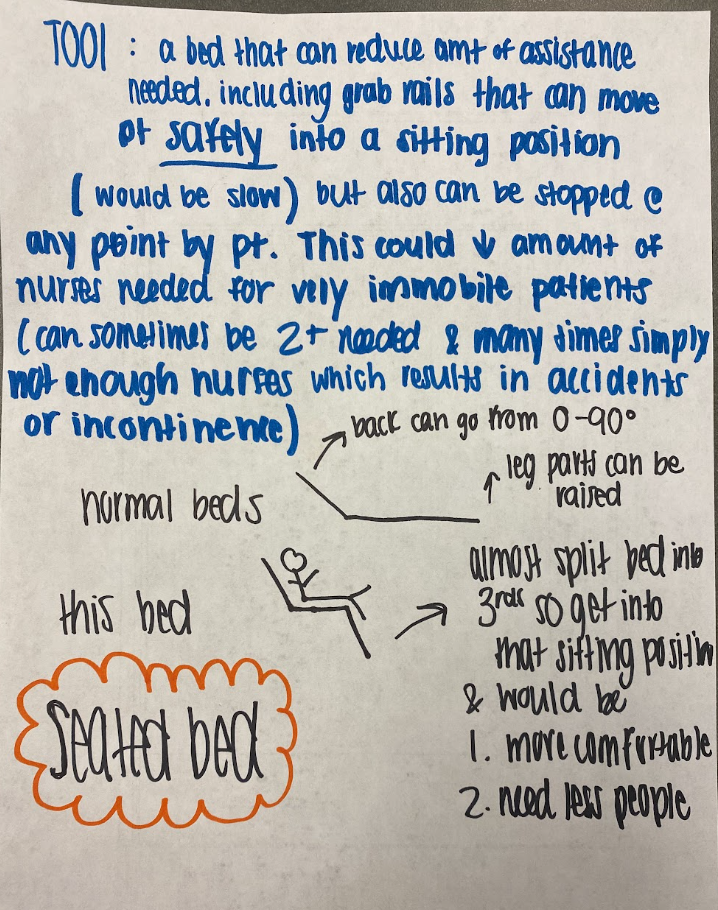
**

Supplemental Figure 6: Design #6: Seated Bed

The sixth design was titled “Seated Bed”. The bed would be broken up into three parts– the upper part of the bed could be moved up to 90 degrees to put the patient in an upright position, the middle part would be a normal “seat,” and the lower part of the bed could be raised or lowered to support the patient’s legs. The seated bed would support the patient’s legs and back the entire time it is used and would reduce the amount of nurses needed to get the patient into the seated position. There would be a pause button as a failsafe that would stop the bed from moving in the event that something goes wrong.

Supplemental Table 6: Design #6: Seated Bed Average Ratings


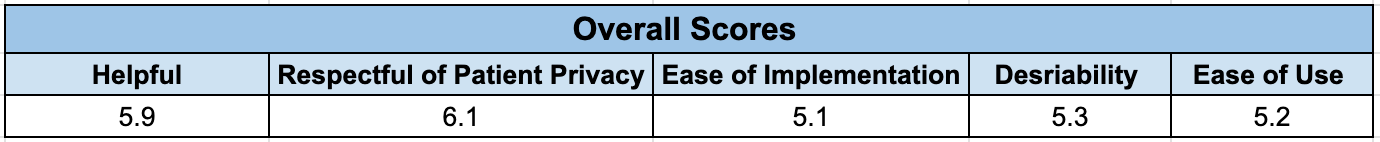


**
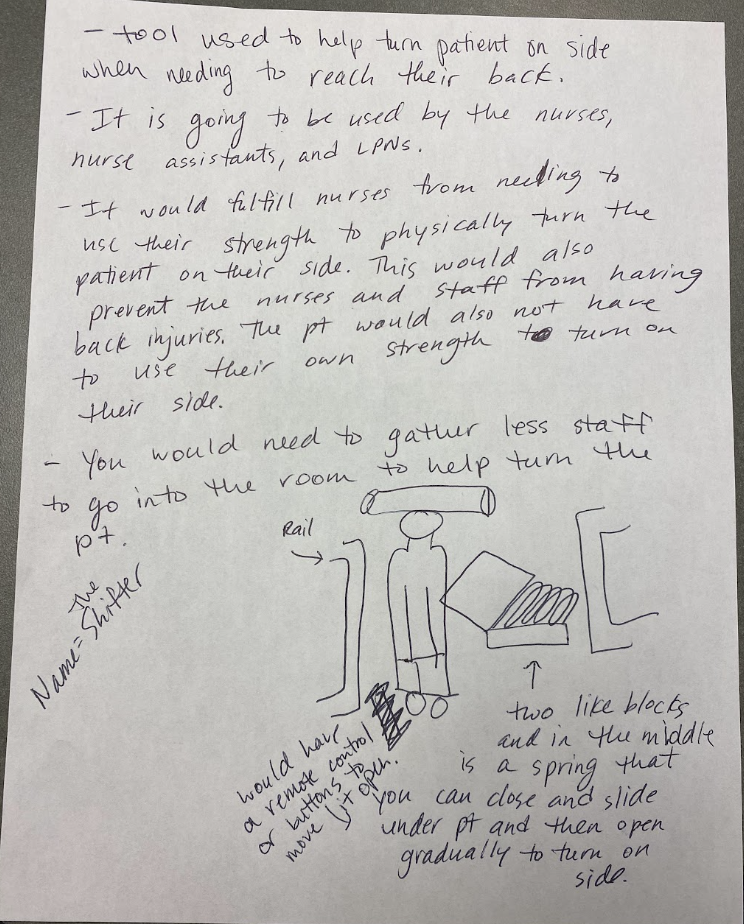
**

Supplemental Figure 7: Design # 7: Shifter

The seventh design was titled “Shifter.” It would help move a patient who is in the bed onto their side, which would expose their backside. This would eliminate the patient’s need to turn. It would be constructed of two metal plates that would be closed together and use a spring or hydraulic system to shift them onto their side.

Supplemental Table 7: Design # 7: Shifter Average Ratings


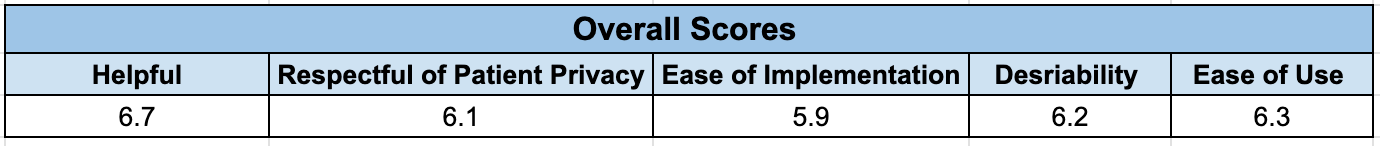


**
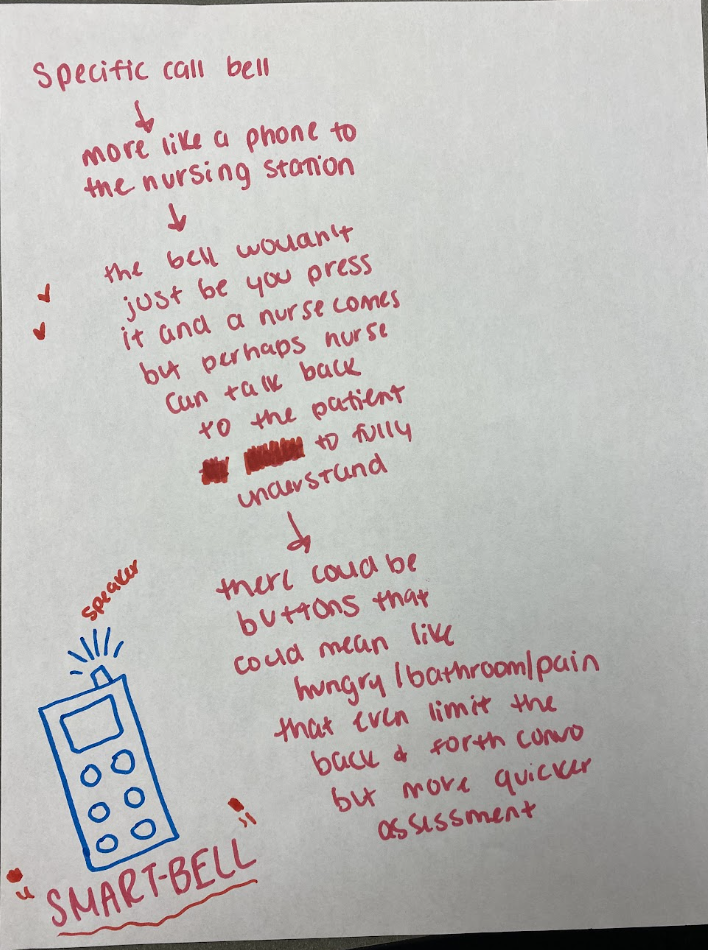
**

Supplemental Figure 8: Design #8: Smart Bell

The eighth design was titled “Smart Bell”. This design has a phone-like function so that the patient would be able to communicate directly with the nurses station. There are also additional buttons for hunger, thirst, etc. This would help the nurse understand what the patient needs and help reduce the number of trips the nurses have to take to help the patient.

Supplemental Table 8: Design #8: Smart Bell Average Ratings
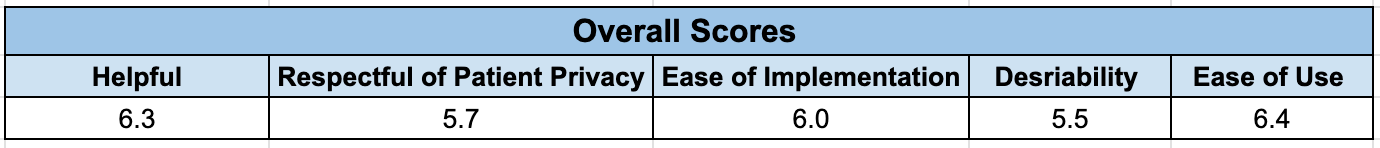


**
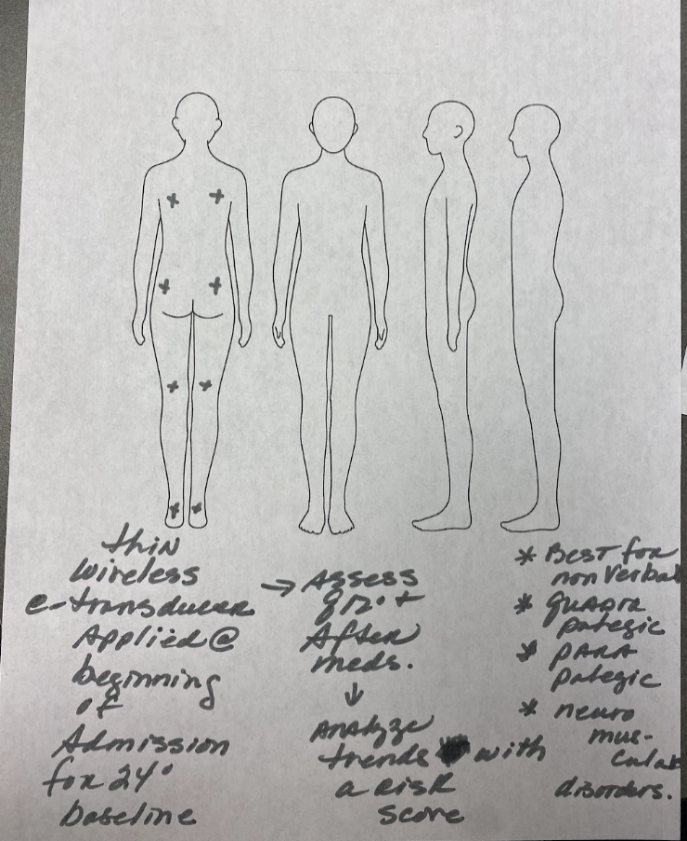

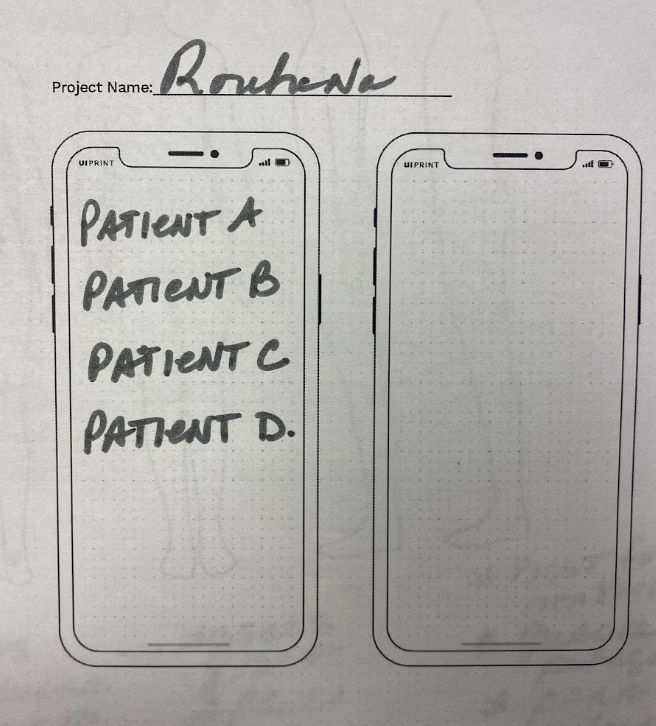
**

Supplemental Figure 9: Design #9: Urotech

The ninth design was titled “Urotech”. This design would attach wireless sensors (thin wireless transducers at mid back, buttocks, heels) to the patient when they are admitted to the hospital. This would help to give a baseline reading of the patient to see how often their bladder fills up and they have to go to the bathroom. The readings would go directly to the nurses phones and monitors to track the patients. It would also notify the nurses when the patient’s bladder is close to full, so they can help the patient before there is an accident. This design would also analyze fall risk scores periodically and determine individual trends of patients. This design would be beneficial for patients with lack of mobility neuromuscular disorder.

Supplemental Table 9: Design #9: Urotech Average Ratings
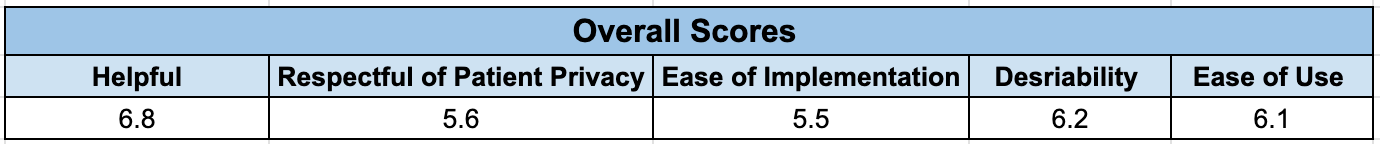


**
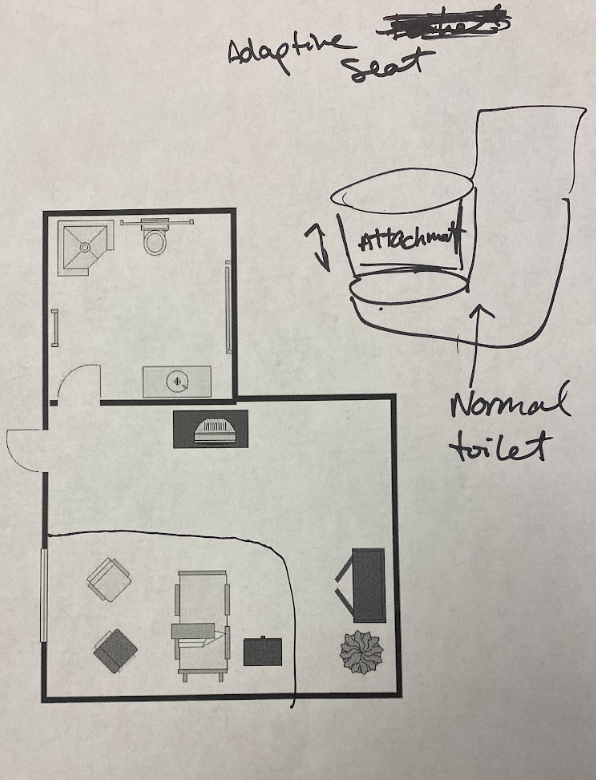
**

Supplemental Figure 10: Design #10: Adaptive Seat

The tenth design was titled “Adaptive Seat.” It involves attachments that could be stacked on top of one another like Legos, and could be attached to either a toilet or commode. The seat would bring the patient to an optimal height that would be most comfortable, and would also be able to tilt in multiple degrees of freedom.

Supplemental Table 10: Design #10: Adaptive Seat Average Ratings
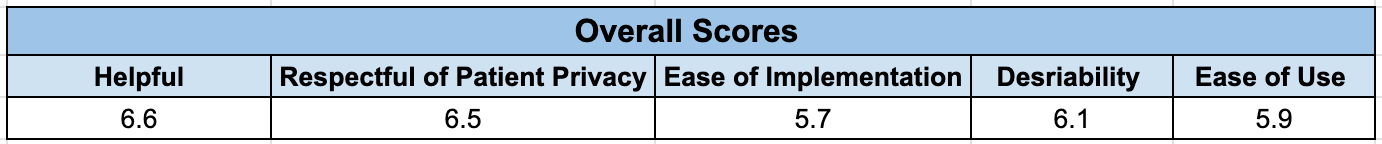


**
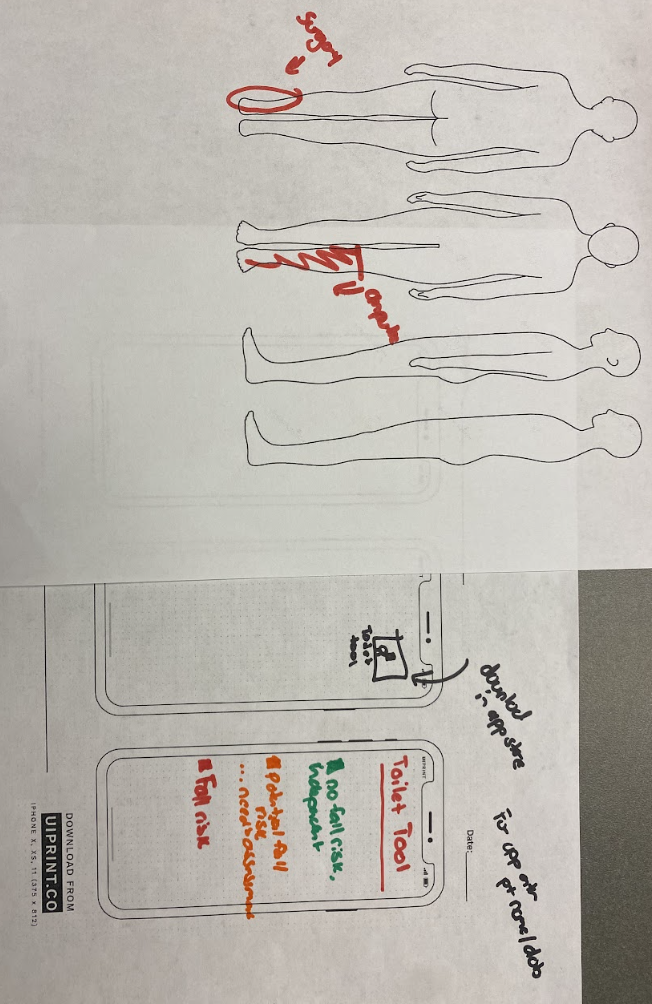

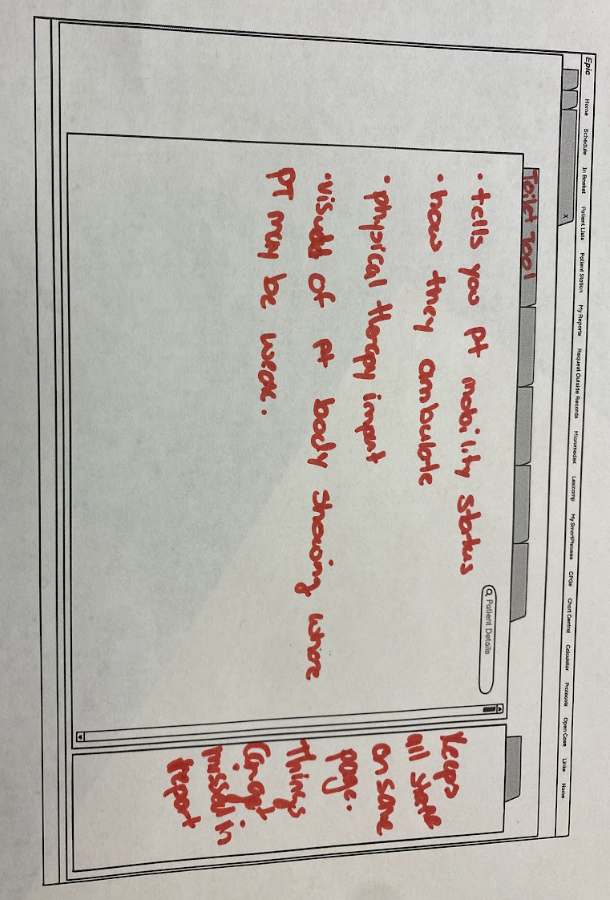
**

Supplemental Figure 11: Design #11: Toilet Tool

The eleventh design was titled “Toilet Tool.” It would involve both a mobile application and a tool that interfaces in the nurse’s computer. It would display the patient’s date of birth, mobility status, how they ambulate, if they have been evaluated by physical therapists, how independent they are, and what languages they know. It would primarily be used for accessing patient data quickly and efficiently.

Supplemental Table 11: Design #11: Toilet Tool Average Ratings


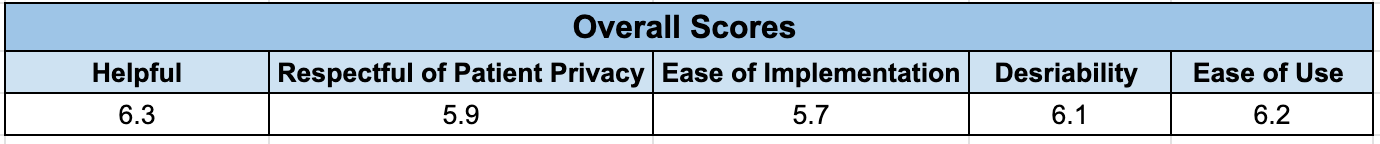

Supplement: Supplementary file 1 [file DataSheet1.DOCX]
